# Application of Magnetic Resonance Imaging in Liver Biomechanics: A Systematic Review

**DOI:** 10.3389/fphys.2021.733393

**Published:** 2021-09-22

**Authors:** Seyed M. Seyedpour, Mehdi Nabati, Lena Lambers, Sara Nafisi, Hans-Michael Tautenhahn, Ingolf Sack, Jürgen R. Reichenbach, Tim Ricken

**Affiliations:** ^1^Institute of Mechanics, Structural Analysis and Dynamics, Faculty of Aerospace Engineering and Geodesy, University of Stuttgart, Stuttgart, Germany; ^2^Biomechanics Lab, Institute of Mechanics, Structural Analysis and Dynamics, Faculty of Aerospace Engineering and Geodesy, University of Stuttgart, Stuttgart, Germany; ^3^Department of Mechanical Engineering, Faculty of Engineering, Boğaziçi University, Istanbul, Turkey; ^4^Faculty of Pharmacy, Istinye University, Istanbul, Turkey; ^5^Department of General, Visceral and Vascular Surgery, Jena University Hospital, Jena, Germany; ^6^Department of Radiology, Charité - Universitätsmedizin Berlin, Corporate Member of Freie Universität Berlin, Humboldt-Universität zu Berlin, and Berlin Institute of Health, Campus Charité Mitte, Berlin, Germany; ^7^Medical Physics Group, Institute of Diagnostic and Interventional Radiology, Jena University Hospital-Friedrich Schiller University Jena, Jena, Germany; ^8^Center of Medical Optics and Photonics, Friedrich Schiller University, Jena, Germany; ^9^Michael Stifel Center for Data-driven and Simulation Science Jena, Friedrich Schiller University, Jena, Germany

**Keywords:** liver, liver disease, biomechanics, magnetic resonance imaging (MRI), elastography, constitutive model, viscoelastic, clinical application

## Abstract

MRI-based biomechanical studies can provide a deep understanding of the mechanisms governing liver function, its mechanical performance but also liver diseases. In addition, comprehensive modeling of the liver can help improve liver disease treatment. Furthermore, such studies demonstrate the beginning of an engineering-level approach to how the liver disease affects material properties and liver function. Aimed at researchers in the field of MRI-based liver simulation, research articles pertinent to MRI-based liver modeling were identified, reviewed, and summarized systematically. Various MRI applications for liver biomechanics are highlighted, and the limitations of different viscoelastic models used in magnetic resonance elastography are addressed. The clinical application of the simulations and the diseases studied are also discussed. Based on the developed questionnaire, the papers' quality was assessed, and of the 46 reviewed papers, 32 papers were determined to be of high-quality. Due to the lack of the suitable material models for different liver diseases studied by magnetic resonance elastography, researchers may consider the effect of liver diseases on constitutive models. In the future, research groups may incorporate various aspects of machine learning (ML) into constitutive models and MRI data extraction to further refine the study methodology. Moreover, researchers should strive for further reproducibility and rigorous model validation and verification.

## 1. Introduction

The liver, an organ weighing between 1.2 and 1.5 kg, depending on age and body mass index (BMI), in humans, is deemed the largest internal organ in adults. It accounts for ~2–5% of the total of body weight (Iwai et al., 2019) and accommodates 25% of the cardiac output (Lautt, 2009). This anatomically and physiologically complex organ (Netter, 2014) is responsible for several functions in the body, including synthesis and metabolism of carbohydrate, protein, and lipid, and detoxification of undesirable substances (Koeppen and Stanton, 2017). Diverse functions of the liver are attributed to a variety of cells of different embryonic origins. These cells are the bile-duct epithelial, stellate, hepatocytes, Kupffer, and sinusoidal endothelial cells and liver stem cells (Trefts et al., 2017). Two blood inflows characterize the vascular system of the liver including the portal vein and the hepatic artery, as opposed to organs such as the kidneys and brain, which are supplied by one inflow of blood. The hepatic blood outflow through hepatic veins are split into intricate tree-like networks upon advancing within the liver (Lebre et al., 2017). The entire liver can be divided into functional units called liver lobules. The liver lobules have a hexagonal structure with the blood inflow at the corners. Blood flows through radially oriented capillaries, the so-called sinusoids, to the centrally located hepatic vein, which drains the blood from the liver. Each liver cell located along those capillaries, interacts with the vascular networks through the sinusoids (Lautt, 2009). The liver shape varies considerably among patients. Distinct geometric and physical properties of liver lobules cause blood circulation through liver lobules to differ among different patients. Consequently, the effects of the geometric characteristics of the liver lobules on intralobular blood flow need to be investigated, which requires an accurate representation of the liver lobules (Ahmadi-Badejani et al., 2020).

Magnetic Resonance Imaging (MRI) and computational methods both play an important role in hepatic hemodynamics studies. Four-dimensional (4D) flow MRI is an adequate tool for assessing the hepatic perfusion, and renders relevant information on spatial and temporal flow, including blood volumes and velocities (Bane et al., 2019). The latter parameters can be used to extract further hemodynamic parameters such as wall shear stress (WSS) (Roldán-Alzate et al., 2013; Wiesemann et al., 2021). Computational methods combined with WSS imaging can provide an adequate view of flow patterns under various anatomical and physiological circumstances, including portal helix and estimation of liver movement patterns (Rutkowski et al., 2019).

Pathological changes in tissues are directly linked to changes observed in the biomechanical properties of tissues such as stiffness (Evans et al., 2010; Shiina et al., 2015) and may occur in the course of various diseases such as carcinoma and malignant tumors (Evans et al., 2020), ectatic corneal disorders (Ford et al., 2011), atheroma, and calcification linked to atherosclerosis (You et al., 2015; Campo et al., 2017), cystic fibrosis-associated liver cirrhosis (Koizumi et al., 2011; Cieciura et al., 2020), and non-alcoholic fatty liver disease (NAFLD) (Mattei and Ahluwalia, 2016). Consequently, the mechanical properties of tissues hold significant diagnostic potential. Surgeons can sometimes diagnose liver tumors that had gone undetected by preoperative imaging, by laparotomy and bare touch (Elias et al., 2005). However, diagnostics via palpation is applicable only in the case of superficial organs. Furthermore, diagnosis by palpation is a subjective examination method that relies on the responsiveness and sensitivity of the practitioner's touch. Regrettably, none of the conventional medical imaging techniques, such as computed tomography (CT), MRI, and ultrasound (US), can demonstrate features that are assessed by palpation. This discrepancy has led to efforts to establish imaging techniques to evaluate the mechanical properties of the tissue quantitatively (Mariappan et al., 2010; Mueller, 2020). For example, the use of liver elastography has led to a considerable reduction in the number of liver biopsies performed to assess liver fibrosis severity (Fang and Sidhu, 2020).

Elastography examines the mechanical properties of biological tissues for diagnostic purposes. In elastography, tissue deformation is externally induced and monitored by encoding tissue motion over space and time within the organ. Various factors, including cellularity, cell types, extracellular matrix deposition, and fluid transport, alter the mechanical properties of biological tissues (Shiina et al., 2015; Sack and Schaeffter, 2018). Many elastography techniques measure stiffness without accounting for the viscoelastic, anisotropic, and nonlinear properties of most biological tissues. Consequently, the associated mechanical properties may change depending on the direction, extent, and rate of deformation. Therefore, the elastic modulus of biological tissues is a complex quantity consisting of a storage modulus and a loss modulus that represent elasticity and viscosity, respectively. The viscosity component shows the damping behavior, in which the strain rate changes with time resulting from the loss of strain energy. Mathematical models such as those proposed by Maxwell, Voigt, Zener, Jeffreys, as well as a fractional Zener model have been devised to predict the stress-strain dynamics of viscoelastic materials (Klatt et al., 2007; Low et al., 2016; Holm, 2019). The elasticity modulus is described per the type of encountered stress as Young's modulus (*E*), Shear modulus (μ), or Bulk Modulus (*K*) (Low et al., 2016). There are different techniques for elastography, all mainly based on US, MRI, and optical coherence tomography methods. In addition to imaging systems, these techniques differ in the source of externally induced stress, which can be static, quasi-static, or dynamic, the latter with the distinction of transient or time-harmonic (Gao et al., 1996; Manduca et al., 2001; Sarvazyan et al., 2011; Wells and Liang, 2011; Vappou, 2012; Low et al., 2016; Ormachea and Parker, 2020; Li et al., 2021). In general, elastography methods apply mechanical stress or stimulation to the tissue, monitor the tissue response to the induced stimulation, and employ this image-encoded response to reconstruct parameters that denote mechanical properties. Meanwhile, elastography techniques are in routine clinical use for the detection of liver fibrosis (Angulo et al., 2007; Yoneda et al., 2007, 2010; Castera et al., 2008; Harrison et al., 2008; Sumida et al., 2011, 2019; Imajo et al., 2016). Other emerging clinical applications of elastography include the diagnosis of cancer (prostate, breast, liver, pancreas) (Thomas et al., 2006; Garteiser et al., 2012; Fischer et al., 2013; Asbach et al., 2020; Zhu et al., 2020), kidney diseases (Garcia et al., 2019; Lang et al., 2019) thyroiditis, arterial plaque evaluation, arterial stiffness (Kolipaka et al., 2012; Schaafs et al., 2019), deep-vein thrombosis evaluation (Hoang et al., 2017; Mumoli et al., 2018), and measurements of human corneal biomechanical properties using optical coherence elastography (OCE) (Wang and Larin, 2015; Lan et al., 2020). Magnetic resonance elastography (MRE) (Muthupillai and Ehman, 1996) is potentially more accurate and less operator dependent than ultrasound elastography (Bonekamp et al., 2009; Faria et al., 2009; Serai et al., 2015; Barr, 2018; Lupescu et al., 2020). One reason for this is the use of time-harmonic mechanical vibrations in a frequency range between 20 and 300 Hz, which can illuminate the full body by shear waves. Furthermore, the excellent soft-tissue image contrast of MRI, which is fully three-dimensional and free of acoustic shading, adds to the high precision and potential accuracy of MRE (Hirsch et al., 2017). MRE uses modified phase-contrast MRI sequences to encode three-dimensional wave fields which are then converted into maps of the complex shear modulus (elastograms) by an inverse algorithm (Venkatesh et al., 2013, 2015; Serai et al., 2015; Tang et al., 2015; Srinivasa Babu et al., 2016; Tzschätzsch et al., 2016; Lupescu et al., 2020; Manduca et al., 2020). The advantages of MRE include the facility to examine the entire liver and convenient application in screening of obese or injured patients (Venkatesh et al., 2013, 2015; Serai et al., 2015; Tang et al., 2015; Srinivasa Babu et al., 2016; Lupescu et al., 2020).

MRI may also serve to define the shape and geometry of the liver in addition to the hemodynamics and the properties of the liver tissue and components. MRI can be applied to reconstruct the liver surface and blood vessels, including portal vein and the branches of the hepatic veins. Moreover, the vein diameters and lengths can be attained from the segmentation data.

The ever-increasing number of MRI-assisted biomechanical studies on the liver underscores the need to integrate existing evidence into research. Systematic reviews address this need by assembling prior research based on standard strategies. Such studies, which are common in medicine (Uman, 2011), may serve as adequate tools for researchers who perform studies in the field of liver biomechanics for gaining a better understanding of the later stages ahead. The main objective of this study is to present a comprehensive overview of current studies on liver biomechanics with the aid of MRI. The secondary objective is to examine the structural models adopted in liver MRE. Furthermore, we intend to propose relevant suggestions for the better use of liver biomechanics in clinical trials.

## 2. Materials and Methods

To design a protocol for the present review, we employed the Cochrane guideline (Higgins et al., 2019) and PRISMA-P checklists (Shamseer et al., 2015) so that the standards of the protocol were satisfied. Although the present review is not of Diagnostic Test Accuracy (DTA) or interventional type (Higgins et al., 2019), the standards complied. Moreover, a modified protocol for implementing review works and evaluating approaches was developed. Given the current review's characteristics, the protocols were modified by making a change in the elements and turning them into research object, object modeling, properties, and tools. The reason is that there are no participant, intervention, comparator, and outcome (PICO) elements available other than in interventional reviews (Higgins et al., 2019). In the latter type of reviews, the research object is described as the tissue or organ under investigation. Imaging techniques, like MRI, CT, and X-Ray, are determined by tools. By object properties, material properties, constitutive relationships, and geometry are meant. In the modeling process, numerical simulation approaches such as the Finite Element Method (FEM), elastic and viscoelastic models for elastography, and the Finite Volume Method (FVM) are implemented. A two-step key term search was employed for finding peer-reviewed research articles related to the topic of the present review. The pre-specified selection criteria were used for designing the basic strategy. As the first criterion, we selected the papers that had employed MRI as their research tool. The second criterion was to select those articles that had investigated the liver from a biomechanical perspective including liver material properties, hemodynamics, and shape and geometry. Although the viscoelastic properties of focal lesions and model identification in liver tumors are extremely interesting, this review focuses on the properties of liver tissue under healthy and chronic disease conditions. We did not exclude liver lesions *per se* from our review, but could not elaborate on this point due to limited data regarding mechanical modeling in liver tumors *in vivo*.

### 2.1. Inclusion Criteria

Based on the elements defined above, the criteria were classified into four classes as follows: The first class includes imaging techniques that are based on MRI, including magnetic resonance imaging “MRI,” magnetic resonance elastography “MRE,” “2D-MRI,” “4D-MRI,” “3D-MRI,” nuclear magnetic resonance imaging “NMRI,” and “DT-MRI.” The second class includes studies that used the terms “Hepatic,” “Liver,” “Liver lobule,” and “Blood perfusion.” The third class contains properties of objects, including “Material property,” “Biophysics,” “Biomechanics,” “Boundary condition,” “Geometry,” and “Mechanical behavior.” The fourth class pertains to numerical plans such as finite difference method “FDM,” “Mesh-free,” finite element method “FEM,” finite volume method “FVM,” computational fluid dynamic “CFD,” “Modeling,” “Simulation,” and artificial neural network “ANN,” which were employed to study the mechanical responses of the liver. As a result, no study was missed due to the constraints on the model types. In this way, we designed a search strategy that retrieved all articles that dealt with liver modeling. Ultimately, the search process was limited to studies that investigated the liver from an engineering point of view. Review papers were not included, only original research papers. To assess all relevant research papers, only papers published during the period 1980–2020 were searched and included. In terms of the used MRI type, no paper was excluded. Only papers in German or English languages were included.

### 2.2. Search Strategy

Using the predetermined selection criteria, we designed an inclusive search strategy for the retrieval of all related articles, and various sources were searched, ensuring that no article was overlooked (cf. Appendix B). Additionally, a list of equivalent terms was prepared and included in the search strategy. For initiating the search, a primary search was performed in the electronic databases on October 25, 2020. Web of Science, Springer, and Medline (Ovid SP) (January 1980–October 2020) were used as search engines. The last search was conducted on December 10, 2020. Moreover, using key terms given in Appendix B, other electronic datasets, such as Wiley, Taylor & Francis, ScienceDirect, and Pubmed were searched. In the next step, a manual search was performed in the references so that any related studies that may not have been found using the search strategy were included.

### 2.3. Selection of Papers

**Figure 1:**
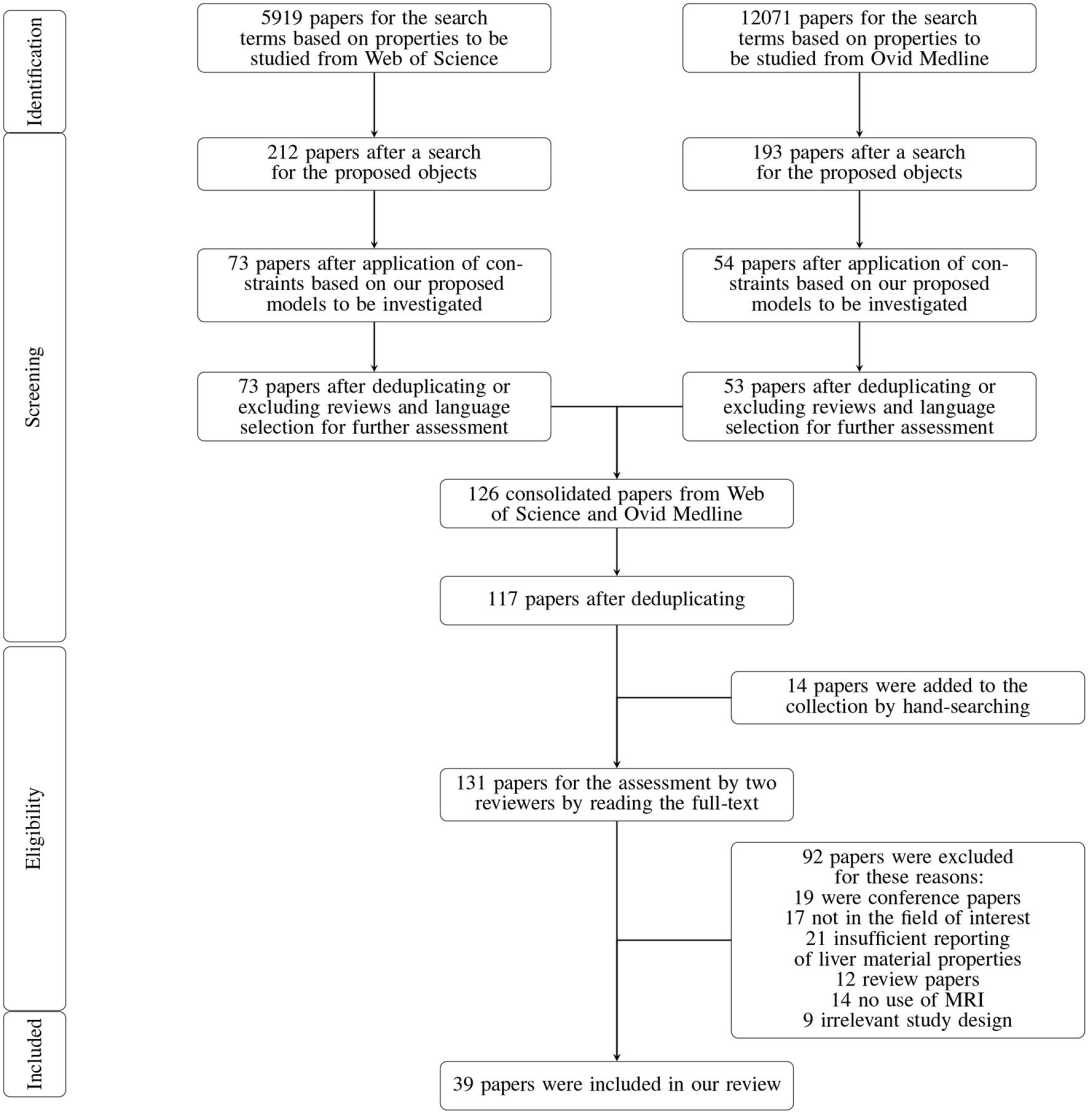
Flowchart of the screening process.

The identified papers were selected based on pre-specified phases. First, papers that were not relevant in terms of titles and abstracts were excluded. Two reviewers assessed the full-text of the remaining papers for obtaining the final list of papers that were included in the review. The flow diagram of the strategy designed for the paper selection is demonstrated in Figure 1.

### 2.4. Extraction of Data

Data were extracted from the included studies by two reviewers, who collected the final data in a previously designed proforma (cf. Appendix C). The data collected in the proforma contained the following properties: The patient or object under study, MRI type, application of MRI, modeling field, the purpose of research, constitutive model, and research clinical application.

### 2.5. Quality Assessment

To assess the quality of the included papers, some questions were designed according to previous methods applied for quality assessment in standard protocols, like PRISMA-P and Cochrane (Shamseer et al., 2015; Higgins et al., 2019). The issues proposed in the standard guideline were modified following this context. Next, questionnaires used in systematic review articles of biomechanics studies (Riva et al., 2013; Hindle et al., 2019) were utilized for preparing a questionnaire (cf. Appendix D). This set of questions includes five main sections, including research objectives, research design, result presentation, bias assessment, and ethical issues. Each question receives a score of 0, 1, 2, or not applicable. Two reviewers separately conducted the assessments. Three groups of items, i.e., the items related to research objectives, research design, and assessment of bias, were regarded as critical as agreed by both reviewers. Thus, the reviewers assessed the quality of the studies considering these items. By definition, a high-quality study was defined as a study that had a maximum of one zero score in the critical items. Studies that received two zero scores on these items and a maximum of one zero score on the non-critical items were considered average-quality papers. Papers with more than or equal to two zero scores in critical items, and more than one zero score in non-critical items were considered papers with low quality.

## 3. Results

### 3.1. Results of the Systematic Search

The search through the electronic databases Ovid Medline and Web of Science resulted in 17,990 papers. The liver was the research object in 405 of the papers. Then, constraints were applied based on the models under study, leading to 127 studies. Review papers, duplicate papers, and papers in languages other than German or English were excluded after applying the limitations in the search platforms, resulting in 117 papers remaining. After inclusion of 14 studies from the hand-search, 131 papers were assessed by two reviewers by reading the full-texts. Eventually, 46 papers were selected for quality assessment and data extraction. The detailed flowchart of paper selection can be seen in Figure 1. Short descriptions of the reviewed papers are presented in Appendix E.

### 3.2. MRI Types and Application

MRI scanners can be classified according to the strength of the magnetic field (McRobbie et al., 2017). Low-field MRIs (0.3 T) are much less expensive, provide better patient access. The main disadvantage of 0.3 T is the reduced signal-to-noise ratio compared with High-field MRI systems (Konar and Lang, 2011). The most common field strength used in clinical applications are 1.5 and 3 T. Assessment of the liver using 1.5 or 3 T MRI has both advantages and disadvantages. The Signal to Noise Ratio (SNR) is higher with 3 T systems, resulting in improved image quality and shorter scan time. Further advantages of 3 T compared to 1.5 T are higher in-plane spatial resolution and thinner slice thicknesses. Almost all of the reviewed papers used 1.5 or 3 T MRI systems, but it is expected that as the availability of 7 T MRIs systems becomes more widespread. Since the SNR of a 7 T system is slightly higher than a 3 T system, and extra SNR leads to higher resolution images (McRobbie et al., 2017). Furthermore, 7 T relaxation values have a slightly more significant difference from 3 T values when assessing differences between healthy and unhealthy liver *in vivo*. Of the 46 studies, 21 used a 1.5 T scanner (Kruse et al., 2000; Brock et al., 2005; Klatt et al., 2007; Salameh et al., 2007; Asbach et al., 2008, 2010; Chen et al., 2011; Wang et al., 2011; Garteiser et al., 2012; Godfrey et al., 2012; Kamphues et al., 2012; Leclerc et al., 2013, 2015; Courtecuisse et al., 2014; Lee et al., 2014; Tzschätzsch et al., 2014; Reiter et al., 2018, 2020; Hudert et al., 2019; Shahryari et al., 2019; Dzyubak et al., 2021), six a 3 T scanner (Roldán-Alzate et al., 2013; Monti et al., 2014; Ning et al., 2018; Rutkowski et al., 2018, 2019; Amili et al., 2019), and four studies a 7 T scanner (Salameh et al., 2009; Riek et al., 2011; Reiter et al., 2014; Ronot et al., 2014). Two studies used both 1.5 and 3 T (Lee et al., 2010; Motosugi et al., 2019), and one study used 0.3 and 3 T (Tomita et al., 2018). Twelve studies (Hariharan et al., 2007; Clarke et al., 2011; Lara et al., 2011; Zhang et al., 2013, 2014; Lu and Untaroiu, 2014; Tang and Wan, 2014; Idkaidek and Jasiuk, 2015; Stoter et al., 2017; Ma et al., 2019; Eaton et al., 2020; Gidener et al., 2020) did not mention the magnetic field strength of the used MRI scanner. Of the 46 studies, six used MRI to determine the geometry and surface of the liver, eight used MRI for hemodynamic studies of the liver, four used MRI for motion and deformation capture of the liver, and 29 used MRI in order to study the elastography and tomoelastography of the liver. Further details of the MRI acquisitions and applications are summarized in Appendix F.

### 3.3. Viscoelastic Models Used in the Liver MRE

A variety of viscoelastic models were used in the liver MRE studies in the reviewed papers. Of the 46 reviewed papers, 29 papers performed MRE on the liver. Four papers studied the liver with linear isotropic elastic models (Kruse et al., 2000; Tang and Wan, 2014; Tzschätzsch et al., 2014; Leclerc et al., 2015), which provide a simple and straightforward relation between stress and strain. Among them, one paper compared the elastic results with the Voigt model (Tzschätzsch et al., 2014) as the simplest viscoelastic model. Four papers used the Voigt model (Klatt et al., 2007; Salameh et al., 2007; Leclerc et al., 2013; Tzschätzsch et al., 2014) and three of them compared the results with the other models. Among these papers, Klatt et al. (2007) compared Voigt, Maxwell, Zener, Jeffereys, and fractional Zener. In three studies, the liver was described with the Zener model (Klatt et al., 2007; Asbach et al., 2008; Tomita et al., 2018), and in four papers the liver was modeled with the spring pot model (Riek et al., 2011; Kamphues et al., 2012; Leclerc et al., 2013; Reiter et al., 2014). One paper studied the liver with an exponential model (Clarke et al., 2011) for large strain, while three papers did not mention their model (Salameh et al., 2009; Garteiser et al., 2012; Ronot et al., 2014). The material models reported in the reviewed articles have been categorized and are listed in Appendix G. MRE at higher field strengths, such as at 3 T, has become a routine application in clinical imaging for the detection of liver fibrosis. In particular, spin-echo MRE sequences are used to mitigate the strong signal T2* decay in the liver at higher field strengths. However, it should be noted that 1.5 T scanners, although increasingly less popular for clinical MRI, are advantageous for liver imaging. Specifically, MRE of livers in the presence of iron overload benefits from less signal decay at 1.5 T than at 3 T. The relatively narrow range of frequencies (10–100 Hz) in MRE is certainly an issue. Therefore, studies relied on simple two-parameter viscoelastic models do not overfit shear modulus dispersion curves. Although two-parameter fits based on a typical set of four frequencies acquired by MRE can significantly vary with noise and data quality, the main tendency of viscoelastic parameter changes has been consistent across studies and etiologies. Furthermore, eight independent measures are fitted by only two parameters if complex modulus data are analyzed, providing sufficient stability for viscoelastic modeling.

### 3.4. Clinical Application

Combining liver biomechanics with MRI techniques could be beneficial in many medical applications.

4D MRI can provide valuable knowledge to understand hepatic hemodynamics, which is important for treating congenital heart defects (Lara et al., 2011). Moreover, the incorporation of circulation in all hepatic vessels indicates a higher potential for clinical use in the hepatic perfusion model (Ma et al., 2019). Computational simulations using a combination of 4D MRI flow and particle image velocimetry can reveal the relationships between portal vessel geometry, flow structure development, and blood flow distribution in distal hepatic vasculature (Rutkowski et al., 2019). In pre-treatment settings, MRI images can be registered using FEM-based deformable image registration, which allows the visualization and contouring of the liver on each image to direct the deformation of other regions of interest. This may be particularly useful for MR registration, where the tumor may have different image characteristics for each modality, and the deformation of the image can compromise geometric accuracy (Brock et al., 2005). Viscoelasticity-based MRE can be a diagnostic tool for detecting the disease process, such as cirrhosis (Asbach et al., 2008), fibrosis (Salameh et al., 2007), or fibrosis in chronic hepatitis C transplant patients (Kamphues et al., 2012), and might become a useful alternative method to liver biopsy. Furthermore, liver elastography might have a role in the early detection and treatment assessment of nonalcoholic steatohepatitis in patients (Salameh et al., 2007). In addition, MRE protocols could be applied for the follow-up of the effects of treatments (Leclerc et al., 2015).

### 3.5. Objects of Reviewed Papers

Of the 46 papers, 30 studied human liver, while 12 dealt with animal liver (Kruse et al., 2000; Hariharan et al., 2007; Salameh et al., 2007, 2009; Clarke et al., 2011; Riek et al., 2011; Courtecuisse et al., 2014; Reiter et al., 2014; Ronot et al., 2014; Tang and Wan, 2014; Idkaidek and Jasiuk, 2015; Ning et al., 2018). Phantoms and experimental models as liver-mimicking material were studied in three papers (Lara et al., 2014; Leclerc et al., 2015; Amili et al., 2019), while two papers investigated both the human liver and phantoms (Lee et al., 2010; Tomita et al., 2018), and one paper studied human and animal liver (Reiter et al., 2014). Thirty-two articles (69.56%) examined liver *in vivo*, while four (8.69%) studied liver *ex vivo*. Eight (17.39%) studies were performed *in vitro*, and two papers reported both *in vitro* and *in vivo* experiments. Eight studies investigated both genders and 11 studies did not mention the gender of the patients. Six studies included only healthy subjects. However, there were 10 studies in which healthy and unhealthy subjects were examined at the same time. Twenty studies investigated liver diseases. Detailed study type, population, and diseases are summarized in Appendix H.

### 3.6. Quality Assessment

In accordance with the protocol mentioned in the Materials and method section, the quality assessment was conducted separately by two reviewers (SMS, MN). The results are summarized in Appendix I. There were 32 high-quality papers, three medium-quality papers, and eleven low-quality papers among the retrieved papers. Of the low-quality papers, seven did not mention any hypotheses and had fewer than three participants. Two of the medium-quality papers had the same problems; however, due to fewer problems with the non-critical criteria, they were ranked medium-quality papers rather than low-quality papers.

## 4. Discussion

### 4.1. Hepatic Hemodynamic Measurement Using 4D Flow MRI

Hemodynamic parameters in splanchnic vessels, such as volume, pressure, velocity, and stress, can presumably depict vascular changes caused by liver diseases such as cirrhosis and portal hypertension, hepatocellular carcinoma, steatosis, and vascular occlusion (e.g., Budd-chiari-Syndrom; Scheinfeld et al., 2009; Zhang et al., 2011; Stankovic et al., 2013). Most hepatic diseases cause a heterogeneous lobar distribution hypothesized due to imbalanced distribution of blood flow between portal venous branches (Lara et al., 2011; Roldán-Alzate et al., 2015). 4D flow MRI has been developed as a beneficial non-invasive tool for the assessment of hepatic hemodynamics (Bane et al., 2019). Furthermore, before and after liver surgery, results of 4D flow MRI provide an insight into hemodynamic changes resulting from resection. The use of 4D flow MRI can serve as a source for simulation conditions and a benchmark for numerical blood flow simulations (Rutkowski et al., 2018). Since the results of pre-surgical 4D flow MRI alone cannot reliably provide information about the liver hemodynamics after surgery, numerical simulations are required to predict the chance of surgical success.

Although 4D flow MRI can measure flow parameters in any vessel contained in the acquired volume by measuring not only a volumetric velocity map but also considering cross-sectional area changes in the vessel of interest during the cardiac cycle, it is challenging because of the need for large volumetric coverage, high spatial resolution, sensitivity to a wide range of flow velocities, the need for respiratory gating and the need for short scan times (Roldán-Alzate et al., 2015). Therefore, the use of cartesian-based 4D flow approaches has been recommended for portal vein research (Stankovic et al., 2014).

Liver stiffness measured by elastography in MRI and ultrasound has been shown to be sensitive to blood flow and perfusion, which can be altered by physiological variations such as water intake and respiratory maneuvers. Because hemodynamic variations are a confounding factor for measured shear stiffness, it is recommended that elastography studies be performed under standardized fasting and breathing conditions to improve the reproducibility and diagnostic accuracy of the method. A model of elastic behavior is developed based on the flow of viscous fluids through the extensive network of tissue microchannels in response to applied stress to study the functional (non-pathological) of the liver (Parker, 2014). Furthermore, the integrated model parameters are directly linked to tissue vascularity and fluid channels in order to predict an abnormal condition (Parker et al., 2016).

### 4.2. Perfusion MRI

Perfusion MRI evaluates the microcirculatory state of the liver parenchyma and lesions, which can be used to identify liver metastases, evaluate antiangiogenic therapy efficacy, determine tumor viability after anticancer therapy or ablation, and diagnose liver cirrhosis and its seriousness (Thng et al., 2010; Hariharan et al., 2013; Stoter et al., 2017). Perfusion MR parameters can be derived using model-free or model-based techniques which analyze the contrast concentration-time curve in focal liver lesions or liver parenchyma, derived from the dynamic contrast-enhanced MRI images (Moawad et al., 2020). The model-free method is based on capturing the rate of tissue enhancement in relation to contrast passage through the tissues. To calculate the hepatic perfusion index, perfusion parameters are derived from the maximal slope of the time-to-intensity curves of the hepatic artery and portal vein (Moawad et al., 2020). The model-based approach includes curve fitting of a dual-input single-compartmental model. The parameters obtained from the model measure the agent's pharmacokinetic distribution in physiological terms such as arterial and portal venous blood flow, distribution volume, and mean transit time. However, it should be mentioned that the model-based methods are based on certain assumptions, which may influence the precision of the derived parameters (Zhou et al., 2020). Moreover, the perfusion profile of the liver, which can be computed with the diffuse interface method, reproduces key properties of the flow, such as regions of higher and lower flow velocities due to local narrowing or widening of vessel cross-sections (Stoter et al., 2017).

### 4.3. Liver Segmentation in MRI

Reliable liver segmentation has particular relevance in the planning (Chlebus et al., 2018), monitoring, and treatment of various liver diseases such as diffuse liver disorders, liver cancer (López-Mir et al., 2014), and liver transplantation (Goldaracena and Barbas, 2019). In comparison to other organs, liver segmentation is more complicated. These complications arise because of the liver's extremely complex structure and adjacency to other tissues. Furthermore, the liver is affected by a variety of pathologies that alter its mass, signal strength, and architecture (Gotra et al., 2017). Liver segmentation strategies include manual, semi-automated, and fully automated. In manual segmentation, the operator performs liver segmentation on sequential MRI slices by contouring pixels around the boundaries or inpainting the liver parenchyma. This approach is strongly dependent on operator interaction. Semi-automated segmentation techniques, such as intensity-based techniques and graph cut, have to be initialized by the operator. The rest of the optimization is performed by the algorithm. These methods often rely on a variety of interactions. For typical datasets, fully automatic segmentation strategies need minimal operator feedback. However, in special cases, they can require manual modification. Since liver segmentation is labor-intensive and time-consuming, deep learning (DL) has been applied for automated liver segmentation (Chen et al., 2020).

### 4.4. Viscoelastic Models in the Liver MRE Studies

**Figure 2:**
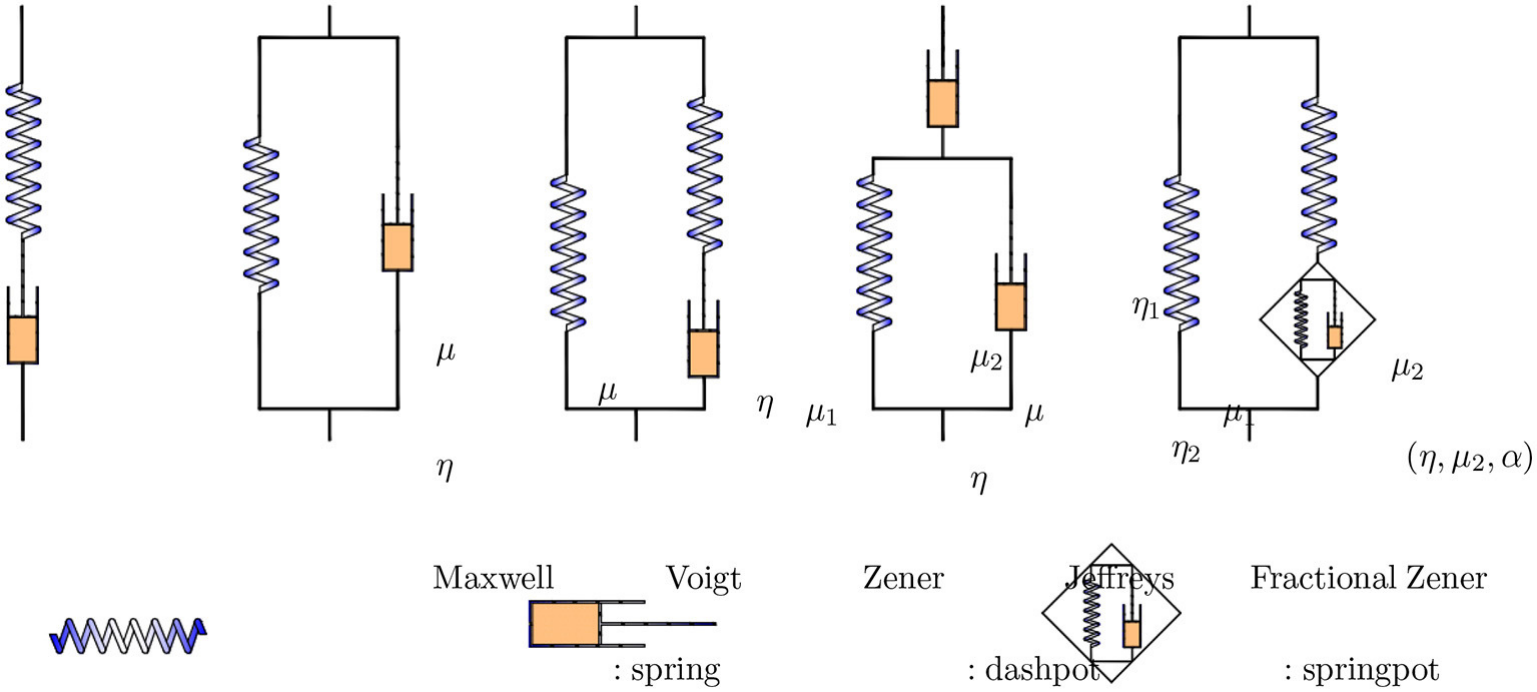
Common viscoelastic models used in liver MRE study.

MRE depends on the stress-strain response of soft tissues exposed to external stimuli. This response can be modeled by combining elastic and viscous elements that characterize the particular rheological behavior of the tissue. Viscoelastic parameters of tissues can be measured using MRE with different frequencies, which enables the deduction of the dispersion of the wave propagation speed and the coefficient of wave-damping (Kruse et al., 2000; Klatt et al., 2007; Sack et al., 2008). MRE data can be used to determine different constitutive parameters of different rheological models, including the models of Voigt, Maxwell, Zener, Jeffreys, and the fractional Zener model (cf. Figure 2). These models consist of three independent constitutive parameters, namely spring, dashpot, and spring pot. The Voigt and Maxwell models both contain a spring and a dashpot. The Zener and Jeffreys models are extensions of the Voigt model. While the Zener model has an additional spring connected in series with the dashpot compared to the Voigt model, the model of Jeffreys has an additional dashpot in series with the Voigt model. The fractional Zener is also an extension of the Zener model, in which a spring pot element replaces the dashpot. The spring pot represents an interpolation between purely elastic and purely viscous behavior, which can be reduced to a spring and a dashpot. The Zener model can better reproduce the dispersion of experimental data, while the fractional Zener model can increase the variation of the results (Sack et al., 2008). The Voigt and Maxwell models are the simplest viscoelastic models. The Voigt model is often used for solids, while the Maxwell model is used for fluids (Catheline et al., 2004). Of the simplest viscoelastic models, the Voigt model tends to be best suited to the viscoelastic parameters of soft tissues (Catheline et al., 2004; Salameh et al., 2007). The Jeffreys model is more suitable in the study of the fluids (Sack et al., 2008; Leclerc et al., 2013). The Zener model can better reproduce the dispersion of experimental data, while the fractional Zener model can increase the variation of the results (Riek et al., 2011). Although the structure geometry parameter α, which is dimensionless power law exponent (cf. Appendix G), does not change with the stage of fibrosis in the liver, μ is the only parameter to be evaluated in order to determine the stage of fibrosis in clinical practice. Hence, the robust spring pot model is especially suitable for the study of liver fibrosis in comparison with the Voigt and Maxwell models (Asbach et al., 2010). More details on the attenuation of shear waves in viscoelastic powerlaw media can be found in Holm (2019).

The constitutive parameters (η, μ, α) can be related to complex modulus G^*^ using various viscoelastic models. G^*^ consists of a real part (G′), which denotes elasticity, and an imaginary part (G^′′^), which denotes viscosity. G′ is determined by the mechanical energy recovery to the material's elastic properties, while G^′′^ is connected to the inherent mechanical friction of the viscous properties given to the tissue (Meyers and Chawla, 2008). The magnitude of the complex shear modulus (|G^*^|) is often referred to as stiffness, but since it represents the geometric mean of (G′) and (G^′′^), it combines both elastic and viscous properties. In this respect, the connotation of (|G^*^|) as stiffness is a colloquial term that conveys the subjective haptic impression of whether a solid tissue with predominantly elastic properties behaves stiffer than surrounding tissues. The phase angle (φ) of G^*^ illustrates fluidity. φ = 0 represents pure solid, while $\varphi =\frac{\pi }{2}$ is representative of pure fluids. $\varphi <\frac{\pi }{4}$ indicates solid dominant tissues, while $\varphi >\frac{\pi }{4}$ illustrates fluid dominant tissues (Hirsch et al., 2017; Shahryari et al., 2019).

Alterations in the hepatic stiffness are associated with changes in solid-tissue properties, and fluid-tissue properties are associated with changes in the stiffness of liver tumors. Hence, fluidity could be a biomechanical marker in order to detect and distinguish liver tumors (Shahryari et al., 2019). Tomoelastography is an *in vivo* method for quantitative mapping of the solid-fluid properties of soft tissues (Hudert et al., 2019; Shahryari et al., 2019). Furthermore, tomoelastography can be used for the evaluation of pediatric NAFLD (Hudert et al., 2019).

Studies using ultrasound-based and MR elastography have shown that the liver stiffens with food and water intake or portal hypertension. Parker (2014) proposed a microchannel flow model which can explain the stiffening of liver tissue due to changes in temperature or salinity of the vascular fluid. Moreover, Parker et al. have theoretically investigated the influence steatosis and steatohepatitis on the liver's viscoelastic properties by simulating composite materials of oil-gelatin mixtures (Parker et al., 2018). The results nicely demonstrate the sensitivity of frequency dispersion of viscoelastic parameters to the fat content in the liver. However, since our review work systematically analyzes viscoelastic modeling of *in vivo* liver tissue, we cannot include phantom investigations without moving the topic of this review article to an entirely new area of focus.

### 4.5. Magnetic Resonance Poroelastography (MRPE)

The shear module achieved by reconstructing the biological soft tissue, based on common MRE, cannot be descriptive of the distribution of true solid matrix parameters (Perriñez et al., 2008). Furthermore, tumors tend to have shear moduli between one and two orders of magnitude higher than surrounding tissue, which can be interpreted as poroelastic material rather than viscoelastic material (Sowinski et al., 2020). Magnetic resonance poroelastography (MRPE) allows the analysis of tissue-poroelastic behavior by distinguishing the mechanical response of the solid matrix from the free extracellular fluid (Perriñez et al., 2008). MRPE determines the time-harmonic pore-pressure field, which is caused by external vibration, while MRE is based on the single-phase elastographic image reconstruction (Perriñez et al., 2010). MRPE can adequately describe the mechanical properties of the liver and its poroelastic deformation. Hence, MRPE could provide more representative mechanical properties of the liver for the simulation of liver disease and liver transplantation. PMRE provides fluid-related quantities, including pore-fluid pressure and hydraulic conductivity (Tan et al., 2017), but it cannot provide pressure boundary conditions (PBCs).

### 4.6. Clinical Application

One of the main objectives of the reviewed studies is how physicians can use the results of MRI-based biomechanical researches as medical diagnostic methods in clinical trials. Although most of the papers included in our review provide a view on the clinical application, not all of them report on the actual applications. Besides, among the studies included in our review, only 12 studies recruited healthy and sick subjects. These studies provide useful information on the value of providing control groups in order to conclude that the procedure in the clinic can be used. Since the clinical use of an intervention depends largely on the quantity of the evidence, the reproducibility of results on a higher number of volunteers would help expand the use of simulations in clinical settings.

### 4.7. Continuum-Biomechanical Models of the Liver

Soft tissues, especially the liver, are materials whose mechanical response is characterized by complex layer heterogeneity, anisotropy, and non-linearity. Although several constitutive models of the liver have been developed, a realistic, comprehensive constitutive model covering the entire spectrum of the liver's complex response is still not available. Improved constitutive models could enable the simulation of the liver deformation and become increasingly valuable in many areas of biomedical engineering. Such constitutive models for the liver, combining advanced imaging and computational biomechanics, can help in medical and industrial development. The liver has been treated as purely macroscopic with a single scale model as linear viscoelasticity material in the earliest models (Liu and Bilston, 2000). Although such a model cannot account for the non-linearity of the mechanical response, the liver can be considered a single-phase viscoelastic material. The effect of fluid pressure on tissue stiffness has been simplified in the effective modulus to describe its time-dependent response. Moreover, the liver has been modeled as nonlinearly hyperelastic (Schwartz et al., 2005). In addition to macroscopic models, multiscale models considered the coupling of the microstructure to the macrovascular structure (Mescam et al., 2009). However, the drawback of single-phase models is that they do not reflect the effect on the microscale of poroviscoelasticity caused by the frictional combination of tissue and blood components. Poroviscoelastic (PVE) models generalize the linear biphasic models that include flow-independent viscoelasticity in the solid-phase description. Different kinds of model approaches exist for fluid-saturated porous solids as the Biot theory (BT) (Malandrino and Moeendarbary, 2019), mixture theory (MT) (Drumheller, 1978; Klisch et al., 2003; Patki and Costanzo, 2019), or Theory of Porous Media (TPM) (de Boer, 2012; Ricken et al., 2015). The BT disadvantage compared to the MT or TPM is its thermodynamic inconsistency and is, therefore, inadequate for further development such as growth.

### 4.8. Liver Diseases Considered in Mathematical Models

Computer-aided calculations have already been developed for a variety of liver diseases. Liver fibrosis is characterized by highly increased accumulation of collagen and thus scarred tissue of the liver. Several models have been presented to describe the development of fibrotic tissue due to various causes, e.g., treatment with carbon tetrachloride (*CCl*_4_) (Dutta-Moscato et al., 2014), chronic Hepatitis B (Wei et al., 2019), and Hepatitis C (Lara et al., 2014) or fibrosis in general (Friedman and Hao, 2017). Cirrhosis is an aggravated and non-reversible form of fibrosis. To investigate perfusion changes in cirrhotic livers, CFD can be used (Peeters et al., 2015). Another method to calculate changes in perfusion in normal, fibrotic, and cirrhotic livers is the porous media approach (Hu et al., 2017). Inherited hepatic diseases can also be studied using computational models. Hemochromatosis, which is caused by an overload of iron in the human body, has been simulated by focusing on the iron metabolism in the liver (Mitchell and Mendes, 2013). Suffering from NAFLD, fat vacuoles are stored in the hepatocytes. This disease, which occurs due to obesity or unhealthy living conditions, has been considered in computational models via homogenization approach on the lobular scale (Ricken et al., 2018; Lambers et al., 2019, 2021; Ricken and Lambers, 2019), simulating fat accumulation and tissue repair (Holzhütter and Berndt, 2020), via ML (Deo and Panigrahi, 2019), focusing on zonated fat accumulation (Ashworth et al., 2016) or to investigate the influence of underlying metabolic processes (Naik et al., 2014; Wallstab et al., 2017; Maldonado et al., 2018). Detoxification of drugs and toxins is one of the main functions of the human liver. Computational models can help to predict detoxification of acetaminophen (Diaz Ochoa et al., 2012; Sluka et al., 2016; Lambers et al., 2018), ammonia and *CCl*_4_ (Schliess et al., 2014). As a consequence of many liver diseases, such as fibrosis or NAFLD, liver cancer can develop by forming tumors in liver tissue. In order to better investigate the development and spread of tumors in the liver, mathematical models can be used. These models primarily differentiate between primary liver tumors such as processes in hepatocellular carcinoma (HCC) (Berndt et al., 2019) and the development of liver tumors through metastases, for example from the colon (Wang et al., 2020). For the identification and prognosis of liver cancer, ML approaches (Keshavarz and Mojra, 2015; Chaudhary et al., 2018; Zhang et al., 2020) are also used, which can map the disease pattern using clinical and experimental data. Mathematical models can also be used to better understand the treatment options for liver tumors. For example, drug-based cancer nanotherapy (Frieboes et al., 2020) or radioembolization dosimetry (Taebi et al., 2020) can be examined for their effectiveness. Although there are already many developed models for the simulation of hepatic processes, some liver diseases are not yet represented by mathematical or numerical models. While some models focus on the mathematical description of hepatitis B and C, no model has yet been developed to describe the underlying processes of hepatitis A. Only models for the epidemiological spread of this disease have been developed to date (van Effelterre et al., 2017). Iberogast®, a widespread over-the-counter herbal product for gastrointestinal complaints, is suspected of having negative effects on liver function and of already being accountable for acute liver failure in one case (Gerhardt et al., 2019). The celandine contained therein is said to be the responsible cause. The liver processes when taking Iberogast®and celandine in general as well as possible predictions and therapies of liver damage have not yet been described mathematically.

### 4.9. MRI-Based Simulations

Several mathematical models based on MRI data have been developed in recent years. To enable a more detailed and patient-specific simulation of hepatic processes, models of the total organ can be coupled with simulations of MRI processes (Mescam et al., 2009). Furthermore, MRI data can be used to include patient-specific information into the models to better represent realistic conditions within the liver. For example, it is possible to simulate hepatic flow based on MRI images in healthy (Stoter et al., 2017; Rutkowski et al., 2019) or cirrhotic patients (Rutkowski et al., 2019). MRI techniques and measurements can be improved with the help of computational modeling, e.g., by using simulations of hepatic blood flow, and different hypotheses for finding new important disease parameters in MRI measurements can be tested *in silico* (George et al., 2015). Since liver transplantation remains the only curative treatment for several liver diseases like malignant liver tumors, a virtual surgery planning helps to improve the construction of hepatectomy in living liver donors during transplantation (Rutkowski et al., 2018). Here, MRI can determine the hemodynamics before and after surgery to validate the surgical planning tool. Also, microscopic hepatic surgeries can be simulated with mathematical models based on MRI data. To combine preoperative data and the resulting deformation of the organ during surgery, computational models can help to improve the liver surgery (Plantefève et al., 2016). Additionally, simulation is used to interpret and analyze MRI data. Therefore, hepatic parameters can be extracted from imaging data and can be used to predict liver function (Forsgren et al., 2014).

### 4.10. Computational Models in Clinical Practice

The main objective of the validated computer-controlled models is their application in clinical practice. To date, there are a few models that are already successfully used by physicians and medical companies to simulate and visualize hepatic processes. Several commercial tools are available to visualize the patient's anatomy using medical images (e.g., Myrian from Intrasense, Ziostation from Ziosoft, Synapse Vincent from Fujinon, Iqqa Liver from Edda Technology, Scout^TM^ Liver from Pathfinder). These programs focus only on medical images neglecting function or perfusion calculation of the liver as well as the individual material behavior. To successfully design a planned operation in advance, programs can be applied to provide a three-dimensional reconstruction of the human anatomy from patient data using virtual reality (Reitinger et al., 2006; Soler et al., 2014) or image segmentation (Schenk et al., 1999; MeVis, 2020). In order to account for changes in the surgical requirements, additional effects such as deformations (Oshiro et al., 2015), material behavior (Marchesseau et al., 2010), and hemodynamics (Rutkowski et al., 2018) can also be taken into consideration.

### 4.11. Strength and Weakness of the Review

Based on our best knowledge, our review is the first systematic review for MRI-based liver biomechanics. Due to the limitations of the search platforms in the engineering fields, we had to base our main search strategy on Ovid Medline and Web of Science, the main search engines in the medical field. While it helped us perform a comprehensive search, this process could not be repeated completely in ScienceDirect, Wiley Online Library, and Taylor & Francis, the fundamental search platforms for engineering fields. Another aspect of our review, which we believe should be seen as a strength and could be a lead for future similar reviews, is using a quality assessment tool based on standard protocols. However, due to the revisions that we had to make, future changes would also be possible. Moreover, future researches will also highlight the need for this kind of assessment, and further revisions will eventually result in standard assessment tools in this field. While one of our objectives was to comment on the best viscoelastic model for various MRE clinical applications, we could not make such recommendations due to the lack of sufficient evidence. Besides proposing the best viscoelastic model for clinical applications of MRE, one of our primary objectives was to assess the quality of the included papers. The results of the assessment, besides a lack of enough evidence for the proposed clinical application, highlight the need for better research designs in the future. A major part of quality assessment is considered to be the population or, in this case, the objects to be studied. Most of the studies were not successful in recruiting enough healthy volunteers or patient subjects for their research, which, therefore, does not represent the target population. To meet the demands of the clinical setting on liver biomechanics, more attention should be paid to participant selection, recruitment, and data collection.

## 5. Conclusion

In this review, recent advances in MRI-based biomechanics for the liver were discussed and summarized. The study focused on the liver MRE, viscoelastic constitutive models and material parameters, and modeling used for liver simulation, the possible clinical applications of these papers, and the studied diseases. Furthermore, the application of liver MRE and simulation for diagnosing liver disease and treatment processes not studied in the papers is discussed. Computational modeling and simulation provide descriptive and predictive tools to identify multiscale interactions and lead to a better understanding of healthy and diseased liver function, potentially in an individualized manner. Despite sophisticated modeling development, liver simulations consistently lag behind in terms of structural knowledge, that is, knowledge of the relationship between structure and function within the liver. The results of liver MRI can fill this gap. This increases the potential to explain the role of the liver and incorporate a technical understanding of the relationship between liver structural changes in diseases. Due to the lack of the best constitutive models for various MRE techniques, researchers may consider the impact of constitutive models on simulating liver disease. In the future, research groups should integrate various ML and MRI data extraction aspects to improve the biomechanical and clinical applications of MRI in liver studies. As a biomechanical application, the artificial neural network (ANN)-based constitutive model possesses the abilities of adjustment, memorization, anticipation, and better performances than the conventional constitutive equations. Therefore, ANNs can provide a novel approach to materials modeling, especially for complex and nonlinear relationships. Furthermore, DL algorithms, including convolutional neural network (CNN), demonstrate strong diagnosis, and grading application of liver diseases such as fibrosis staging, diagnosis of fatty liver, and detection and classification of tumors. In addition, researchers must also seek to achieve reproducibility, validation, and verification of robust models.

## Data Availability Statement

The original contributions presented in the study are included in the article/Supplementary Material, further inquiries can be directed to the corresponding author/s.

## Author Contributions

SS devised the project, the main conceptual ideas, and proof outline. H-MT, IS, JR, and TR helped supervise the project. SS and MN worked out review of the papers with the help from SN and LL. SS wrote the manuscript with input from all authors. All authors provided critical feedback and helped shape the research, analysis and manuscript.

## Funding

This work was funded by Deutsche Forschungsgemeinschaft (DFG, German Research Foundation) under Germany's Excellence Strategy—EXC 2075—390740016, the Research Unit Programme FOR 5151 QuaLiPerF (Quantifying Liver Perfusion—Function Relationship in Complex Resection—A Systems Medicine Approach) by grant number 436883643 and the SPP 2311 SIMulation supported LIVer Assessment for donor organs (SimLivA) by grant number 465194077.

## Conflict of Interest

The authors declare that the research was conducted in the absence of any commercial or financial relationships that could be construed as a potential conflict of interest.

## Publisher's Note

All claims expressed in this article are solely those of the authors and do not necessarily represent those of their affiliated organizations, or those of the publisher, the editors and the reviewers. Any product that may be evaluated in this article, or claim that may be made by its manufacturer, is not guaranteed or endorsed by the publisher.
