# Supplementary material for: Application of Magnetic Resonance Imaging in Liver Biomechanics: A Systematic Review
Source: Front Physiol. 2021 Sep 22;12:733393. doi: 10.3389/fphys.2021.733393 (PMC8493836; doi:10.3389/fphys.2021.733393)
Supplement: Supplementary file 1 [file Data_Sheet_1.pdf]

Supplementary Material

APPENDIX

Appendix A: Summary of the main abbreviations and acronyms used in this paper

Table S1: List of abbreviations and acronyms

Expression	Description
2D	Two-dimensional
3D	Three-dimensional
4D	Four-dimensional
ANN	Artificial neural network
BT	Biot theory
CFD	Computational fluid dynamics
CT	Computed tomography
DTA	Diagnostic test accuracy
DT-MRI	Diffusion tensor magnetic resonance imaging
FDM	Finite difference method
FEM	Finite element method
FVM	Finite volume method
ML	Machine learning
MRI	Magnetic resonance imaging
MRE	Magnetic resonance elastography
MRPE	Magnetic resonance poroelastography
MT	Mixture theory
NAFLD	NAFLD
NMRI	Nuclear magnetic resonance imaging
PBCs	Pressure boundary conditions
PVE	Poroviscoelastic
SNR	Signal to noise ratio
TPM	Theory of porous media
US	Ultrasound
WSS	Wall shear stress

Appendix B: Search strategy for Medline Ovid SP

1. image$ tech$.mp.
2. magnetic resonance imaging.mp.
3. MRI.mp.
4. MRE.mp.
5. magnetic resonance elastography.mp.
6. 2D-MRI.mp.
7. 3D-MRI.mp.
8. 4D-MRI.mp.
9. diffusion tensor magnetic resonance imaging.mp.
10. DT-MRI.mp.
11. diffusion magnetic resonance imaging.mp.
12. diffusion tensor imaging.mp.
13. NMRI.mp.
14. nuclear magnetic resonance imaging.mp.
15. or/1-14
16. liver.mp.
17. blood perfusion.mp.
18. liver lobule.mp.
19. or/16-18
20. biomechanic$.mp.
21. material propert$.mp.
22. biophysic$.mp.
23. mechanical behavior.mp.
24. boundary condition.mp.
25. geometr$.mp.
26. or/20-25
27. 15 and 26
28. 19 and 27
29. FEM.mp.
30. finite element.mp.
31. FEA.mp.
32. or/29-31
33. 28 and 32
34. finite difference.mp.
35. FDM.mp.
36. 34 or 35
37. 36 and 28
38. meshfree.mp.
39. 28 and 38
40. meshless.mp.
41. 28 and 40
42. simulation.mp.
43. 28 and 42
44. modeling.mp.

45. 28 and 44
46. CFD.mp.
47. computational fluid dynamics.mp.
48. 46 or 47
49. 28 and 48
50. 28 and 48
51. ANN.mp.
52. artificial neural network.mp.
53. 51 or 52
54. 28 and 53
55. 33 or 37 or 39 or 41 or 43 or 45 or 49 or 54

Appendix C: The designed proforma for data extraction

Table S2: Designed proforma for data extraction

Title	Aim of the study	Exclusion reason	Subject	MRI type	MRI application	Numerical method	Constitutive model	Clinical application

Appendix D: Quality analysis form

Table S3: Quality analysis form

#	**Question and scoring procedure**
1	Has the study aim been stated?<br>For Yes the score would be 1 and for No the score would be 0.
2	Did the study have a clear hypothesis?<br>For Yes the score would be 1 and for No the score would be 0
3	Has the object selection of the study introduced bias?<br>For score 1, the rational for object selection has been stated.<br>For score 0, the rational for object selection has not been stated.
4	Did the study explain the characteristics of the objects?<br>Two characteristics are considered for scoring: gender and age<br>For score 2, 2 parameters should have been mentioned.<br>For score 1, 1 parameter have been mentioned.<br>For score 0, no parameter has been mentioned.<br>NA for animals and phantoms.
5	Could the number of objects be representative of the intended population?<br>For score 2, at least 5 objects were studied.<br>For score 1, 3 and 4 objects were studied.<br>For score 0, less than 3 objects were studied.
6	Has the study stated ethical approval for<br>animal handling and/or data collection for human objects?<br>For Yes the score would be 1 and for No the score would be 0.<br>NA for the experimental models and phantoms.
7	Did the study describe the MRI function in detail?<br>For Yes the score would be 1 and for No the score would be 0.
8	Has the study described its clinical applications?<br>For Yes the score would be 1 and for No the score would be 0.
9	Did the study describe the used models for MRE or simulation?<br>For Yes the score would be 1 and for No the score would be 0.<br>NA If the paper is not a MRE study.
10	Have the limitations of the study been mentioned?

	For Yes the score would be 1 and for No the score would be 0.
11	Was the study funding mentioned?<br>For Yes the score would be 1 and for No the score would be 0.
12	Has the study stated conflicts of interest?<br>For Yes the score would be 1 and for No the score would be 0.

Appendix E: Short descriptions of included studies

Table S4: Short descriptions of included studies

Author	Short description
(Amili et al., 2019)	The study leveraged a combination of optical and medical imaging techniques, ultimately validating a novel approach to track virtually released particles in volumetric velocity fields.
(Asbach et al., 2008)	In the study, the multifrequency MRE method was used to measure parameters for a viscoelastic model of a liver based on two shear moduli and one viscosity parameter. The investigated viscoelastic parameters show a significant difference between the normal and cirrhotic livers.
(Asbach et al., 2010)	The study analyzed the dynamics of the shear modulus evaluate the optimum driving frequency and to determine the diagnostic accuracy of generalized frequency-independent elasticity cutoff values for staging hepatic fibrosis.
(Brock et al., 2005)	In this study, a platform was developed to perform multi-organ deformable image registration using finite element modeling. Its feasibility and accuracy were demonstrated by deformable image registration of MR images at different respiratory states for both thorax and the abdomen.
(Chen et al., 2011)	The study investigated the diagnostic accuracy of MRE for the early detection of nonalcoholic steatohepatitis among patients with nonalcoholic fatty liver disease.
(Clarke et al., 2011)	The study presented storage and loss moduli data and predictive models for in vitro bovine liver blocks using MRE, under various levels of compressive preload. Moreover, the study describes a device and methods capable of measuring the viscoelastic properties of tissues at large strains in vitro.

Table S4: Short descriptions of included studies in the paper

Author	Short description
(Courtecuisse et al., 2014)	The study combined dynamic image sequence with a physics-based simulation to obtain a 3D representation of the liver during respiration. A pre-operative CT scan of a liver was acquired and it was aimed to register the segmented model to 2D dynamic slices acquired with MRI.
(Dzyubak et al., 2021)	The study hypothesized that widely available rapid MRI techniques could be used to predict nonalcoholic steatohepatitis noninvasively by measuring liver stiffness with magnetic resonance elastography (MRE) and liver fat with chemical shift-encoded MRI. Besides, the study validates an automated image analysis technique to maximize the utility of these methods.
(Eaton et al., 2020)	The study examined the associations between changes to measure hepatic stiffness and primary sclerosing cholangitis. The study showed that hepatic decompensation was independently and robustly linked to the baseline and to changes in liver stiffness over time. While the progression in hepatic stiffness accelerates if hepatic stiffness is high, the overall rate of changes in hepatic stiffness is slow. Therefore, it may be useful to exclude individuals with low baseline liver rigidity if liver rigidity is used as the primary substitute endpoint in early-stage clinical trials.

Table S4: Short descriptions of included studies in the paper

Author	Short description
(Garteiser et al., 2012)	The study assessed the value of viscoelastic parameters in characterizing liver tumors with MRE. The authors found that the loss modulus was the best discriminator between benign and malignant tumors and the only biomechanical parameter that differed between individual tumor types.
(Gidener et al., 2020)	The study aimed to investigate the role of MRE in the prediction of hard outcomes in NAFLD. The study showed that in NAFLD, liver stiffness measurement by MRE is a significant predictor of the future development of cirrhosis. Their data expand the role of MRE in clinical practice beyond the estimation of liver fibrosis and provide important evidence that improves individualized disease monitoring and patient counseling.
(Godfrey et al., 2012)	The study assessed the diagnostic accuracy of MRE stiffness values and the ratio of phosphomonoesters /phosphodiesters measured using ^{31}P spectroscopy against histological fibrosis staging.
(Hariharan et al., 2007)	The study analyzed radio frequency heating in the case of a tumor located near the bifurcation point of a hepatic artery using geometry reconstructed from MRI images of a porcine liver. Moreover, to study the range of influence of blood flow through the bifurcated artery on tissue heating, different tumor locations were considered.
(Hudert et al., 2019)	In this study, multifrequency MRE was used to quantify liver steatosis and fibrosis in adolescents with NAFLD.

Table S4: Short descriptions of included studies in the paper

Author	Short description
(Idkaidek and Jasiuk, 2015)	In this study, a fast and accurate three-dimensional simulation of the deformation of a pig liver under pressure was presented from a surgical tool using ABAQUS. The liver geometry was obtained using MRI, and a nonlinear constitutive law was employed to capture large deformations of the tissue.
(Kamphues et al., 2012)	The aim of this study was to prospectively assess the diagnostic accuracy of viscoelasticity-based MRE for the assessment of liver fibrosis in hepatitis C patients after liver transplantation.
(Klatt et al., 2007)	The study presented an experiment combining multifrequency shear wave actuation with broad-band motion sensitization to extend the dynamic range of a single MRE examination. The technique was applied to the brain and liver of five healthy volunteers, and five standard rheological models (Maxwell, Voigt, Zener, Jeffreys, and fractional Zener model) were assessed for their ability to reproduce the observed dispersion curves. The study found significant differences between the rheological parameters of brain and liver indicating that human brain is softer and has a higher viscosity than liver.

Table S4: Short descriptions of included studies in the paper

Author	Short description
(Kruse et al., 2000)	In this study, fresh animal liver and kidney tissue specimens were evaluated with MRE at multiple shear wave frequencies to investigate the effect of specimen temperature and orientation on stiffness measurements was studied in skeletal muscle. The purpose of the study was to conduct preliminary studies to define methods for using MRE as a tool to address the lack of quantitative tissue mechanical property data in the literature.
(Lara et al., 2011)	The study characterized the flow dynamics of multi-inlet patient-specific pediatric hepatic venous junctions and incorporated transparent rapid-prototype replicas of two pediatric hepatic venous confluence anatomies and two-component particle image velocimetry to investigate the primary flow structures influencing the inferior vena cava outflow.
(Leclerc et al., 2013)	In this study, the relevance of viscosity measurements as a liver diagnostic marker. The variation of the liver viscosity parameter as a function of post-processing revealed that this parameter should be further investigated to demonstrate its relevance in clinical practice.
(Leclerc et al., 2015)	In this study, a 3D FEM phantom model, with realistic MRE liver boundary conditions was developed to simulate the shear wave propagation with the software ABAQUS and to identify the method for the mechanical characterization of phantom mimicking soft tissue.
(Lee et al., 2010)	In this study, a dynamic 3D liver surface instantiation and localization scheme was developed to enable subject-specific optimal scan planning.

Table S4: Short descriptions of included studies in the paper

Author	Short description
(Lee et al., 2014)	The study determined the reproducibility of MRE and the reproducibility and repeatability of the stiffness measurement of MRE in the staging of liver fibrosis.
(Lu and Untaroiu, 2014)	The study established a standard procedure to quantify the shape variations of a human liver in a sitting posture and construct three-dimensional statistical shape boundary models.
(Ma et al., 2019)	The study modeled the hepatic perfusion in a physiologically based subject-specific hepatic structure of a healthy individual. The structured tree boundary condition was implemented for the first time in a computational model of hepatic perfusion, which led to physiologically reasonable results in the blood flow simulation in the hepatic artery and portal vein.
(Monti et al., 2014)	The study designed a computer simulation to reproduce a quantification model of cardiac-induced strain in the liver using tagged MRI. Additionally, it evaluated the performance of the harmonic phase image analysis method and its dependence on fine-tuning of the tag spacing and grid angle parameters currently selected in a heuristic way.
(Motosugi et al., 2019)	The study assessed the feasibility of 4D flow MRI as a noninvasive imaging marker to stratify the risk of variceal bleeding in patients with liver cirrhosis.
(Ning et al., 2018)	The aim of this study was to present a simple method to correct vascular input function due to inflow effects and to test whether the proposed method can provide more accurate vascular input functions for improved pharmacokinetics modeling.

Table S4: Short descriptions of included studies in the paper

Author	Short description
(Reiter et al., 2014)	This comprehensive report described the correlation between static Young's modulus, viscoelastic power-law constants and structural and functional variables of liver tissue to assess the merits of hepatic elastography as a structure sensitive modality.
(Reiter et al., 2018)	The study evaluated and compared the applicability of different elastography methods to assess alpha1-antitrypsin-deficiency related liver fibrosis.
(Reiter et al., 2020)	The study determined the diagnostic performance, cut-off values, and optimal drive frequency range for staging hepatic fibrosis using tomoelastography of multifrequency MRE of the liver and spleen.
(Riek et al., 2011)	The study presented data of $\mathbf{G}^{\star}$ of agarose gel, liver, brain, and muscle samples measured with high-resolution MRE in a 7 T animal scanner at 200–800 Hz vibration frequency. The study aimed to investigate the complex modulus dispersion of tissue samples.
(Roldán-Alzate et al., 2013)	The study implemented and validated in vivo radial 4D flow MRI to quantify blood flow in the hepatic arterial, portal venous, and splanchnic vessels of healthy volunteers and patients with portal hypertension.
(Ronot et al., 2014)	This study investigated which viscoelastic parameter has the best diagnostic performance for quantifying liver fibrosis by 3D multifrequency MRE in a high-resolution model from rat thin liver sections.
(Rutkowski et al., 2018)	In this study, MRI, CFD modeling and in vitro experiments was used to predict patient-specific alterations in hepatic hemodynamics in response to partial hepatectomy in living liver donors.

Table S4: Short descriptions of included studies in the paper

Author	Short description
(Rutkowski et al., 2019)	The study examined the effects of varying spleno-mesenteric confluence anatomy on blood flow distribution and helical flow patterns in the portal vein using 4D flow MRI data from liver donors with computational tools to simulate hemodynamic outcomes from a variety of portal confluence orientations.
(Salameh et al., 2007)	The study determined the correlations between the viscoelastic parameters of the liver measured with in vivo MRE and quantitative analysis of liver fibrosis.
(Salameh et al., 2009)	The study assessed the potential value of MRE imaging to help detect non-alcoholic steatohepatitis in the fatty rat liver.
(Shahryari et al., 2019)	The study used tomoelastography to investigate whether solid–fluid properties can differentiate hepatic tumors from nontumorous liver tissue and malignant from benign lesions.
(Stoter et al., 2017)	The study presented a diffuse interface method for coupling free and porous-medium-type flows modeled by the Navier–Stokes and Darcy equations. Moreover, it demonstrated the method's potential to establish seamless imaging through analysis workflows by computing a perfusion profile for a full-scale 3D human liver based on MRI scans.
(Tang and Wan, 2014)	The study presented a novel strain-based constraint finite-element method for simulating nonlinear homogeneous soft tissues efficiently. The algorithm is capable of modeling rich nonlinear deformations in a straightforward finite-element framework.

Table S4: Short descriptions of included studies in the paper

Author	Short description
(Tomita et al., 2018)	In the study, a finite element model for MRE, which includes the Zener model for the displacement field of a wave in tissue and an inversion algorithm, the so-called modified integral method, was developed using ANSYS.
(Tzschätzsch et al., 2014)	In this study, time-harmonic elastography of the liver was introduced and applied to a group of healthy volunteers in comparison with multifrequency MRE at identical harmonic vibration frequencies.
(Wang et al., 2011)	The study compared the utility of MRE and diffusion-weighted imaging in characterizing fibrosis and chronic hepatitis in patients with chronic liver diseases.
(Zhang et al., 2013)	The study proposed a method for reconstructing 3D dense deformable motion from sparse surrogate motion tracked via on-board imaging systems with the help of a patient-specific principal component analysis motion model.
(Zhang et al., 2014)	The study simulated the use of Beams-Eye-View surrogate imaging along with the motion models, to study the potential effectiveness of scanned beam tumor tracking.

Appendix F: Details of used MRI and their application

Table S5: Details of used MRI and their application

Author	MRI magnetic field	Description	Application
(Amili et al., 2019)	3 T	Siemens Prisma whole-body scanner	Velocity field measurement in a designed hepatic arterial system
(Asbach et al., 2008)	1.5 T	Magnetom Sonata, Siemens Medical Solutions, Erlangen, Germany	Elastography
(Asbach et al., 2010)	1.5 T	Magnetom Sonata, Siemens Healthcare Sector, Erlangen, Germany	Elastography
(Brock et al., 2005)	1.5 T	Excite, 4 channel, GE Medical Systems, Milwaukee, WI	Multi-organ deformable registration
(Chen et al., 2011)	1.5 T	GE Healthcare, Milwaukee, WI	Elastography
(Clarke et al., 2011)	NM	NM	Elastography
(Courtecuisse et al., 2014)	1.5 T	MAGNETOM®Aera SIEMENS	Capture the respiratory motion
(Dzyubak et al., 2021)	1.5 T	GE Healthcare, Milwaukee, WI	Elastography
(Eaton et al., 2020)	NM	NM	Elastography
(Garteiser et al., 2012)	1.5 T	Intera, Philips Medical Systems, Best, The Netherlands	Elastography
(Gidener et al., 2020)	NM	NM	Elastography
(Godfrey et al., 2012)	1.5 T	General Electric whole body system (HDx, GEHT, Waukesha, WI)	Elastography
(Hariharan et al., 2007)	NM	NM	Geometry
(Hudert et al., 2019)	1.5 T	Siemens, Magnetom Sonata	Tomoelastography
(Idkaidek and Jasiuk, 2015)	NM	NM	Geometry
(Kamphues et al., 2012)	1.5 T	Magnetom Sonata, Siemens Healthcare Sector, Erlangen, Germany	Elastography

Table S5: Details of used MRI and their application

Author	MRI magnetic field	Description	Application
(Klatt et al., 2007)	1.5 T	Magnetom Sonata, Siemens Medical Solutions, Erlangen, Germany	Elastography
(Kruse et al., 2000)	1.5 T	NM	Elastography
(Lara et al., 2011)	NM	NM	Geometry
(Leclerc et al., 2013)	1.5 T	GE, Milwaukee, WI	Elastography
(Leclerc et al., 2015)	1.5 T	GE, Milwaukee, WI	Elastography
(Lee et al., 2010)	1.5 T, 3 T	GE, Discovery MR750, Philips Intera	Liver surface instantiation and localization
(Lee et al., 2014)	1.5 T	whole-body MR scanner (SignaHDx; GE Healthcare, Milwaukee, WI)	Elastography
(Lu and Untaroiu, 2014)	NM	NM	Geometry
(Ma et al., 2019)	NM	MAGNETOM Avanto, Siemens, Germany	Visualization of hepatic blood flow and bile flow
(Monti et al., 2014)	3 T	NM	Measurement of the liver stiffness
(Motosugi et al., 2019)	1.5 T, 3 T	Optima MR450w or Signa HDxt, GE Healthcare, Waukesha, Wis Discovery750, GE Healthcare	Flow and velocity measurement
(Ning et al., 2018)	3 T	Discovery MR750, GE Healthcare, Waukesha, Wisconsin, USA	Flow and velocity measurement
(Reiter et al., 2014)	7 T	Bruker Pharmascan, Ettlingen, Germany	Elastography
(Reiter et al., 2018)	1.5 T	Magnetom Aera, Siemens Healthcare, Erlangen, Germany	Elastography
(Reiter et al., 2020)	1.5 T	Magnetom Aera, Siemens Healthineers	Tomoelastography
(Riek et al., 2011)	7 T	Bruker PharmaScan 70/16, Ettlingen, Germany	Elastography

Table S5: Details of used MRI and their application

Author	MRI magnetic field	Description	Application
(Roldán-Alzate et al., 2013)	3 T	Discovery MR 750, GE Healthcare, Waukesha, WI	Quantify flow in the hepatic and splanchnic vasculature
(Ronot et al., 2014)	7 T	Pharmascan, Bruker, Erlangen, Germany	Elastography
(Rutkowski et al., 2018)	3 T	Discovery MR 750, GE Healthcare, Waukesha, WI	Velocity mapping
(Rutkowski et al., 2019)	3 T	Discovery MR 750, GE Healthcare, Waukesha, WI	Determination of blood flow dynamic in liver
(Salameh et al., 2007)	1.5 T	Gyroscan Intera whole body imager, Philips Medical Systems, Best, The Netherlands	Elastography
(Salameh et al., 2009)	7 T	Pharmascan, Bruker, Ettlingen, Germany	Elastography
(Shahryari et al., 2019)	1.5 T	Magnetom Aera, Siemens	Tomoelastography
(Stoter et al., 2017)	NM	NM	Measurement of the liver perfusion
(Tang and Wan, 2014)	NM	NM	Geometry
(Tomita et al., 2018)	0.3 T, 3 T	Signa HDx, GE Healthcare, the Compact MRI series, MR Technology, Inc., Tsukuba, Japan	Elastography
(Tzschätzsch et al., 2014)	1.5 T	Magnetom Sonata, Siemens Erlangen, Germany	Elastography
(Wang et al., 2011)	1.5 T	Magnetom Espree, Siemens Healthcare	Elastography
(Zhang et al., 2013)	NM	NM	Motion extraction
(Zhang et al., 2014)	NM	NM	Motion extraction

NM: Not mentioned

Appendix G: MRE

Table S6: MRE models description

Author	Techniques	Model	Measured parameter
(Asbach et al., 2008)	Multifrequency MRE with frequencies of 25.0,37.5, 50.0, and 62.5 Hz	Standard linear solid (SLS) or Zener:<br>$\mathbf{G}^\star = \frac{\mu_1\mu_2 + \mathrm{i}\omega\eta(\mu_1+\mu_2)}{\mu_2 + \mathrm{i}\omega\eta}$	Normal liver:<br>$\eta = 7.3{\pm}2.3$ (Pa.s), $\mu_1 = 1.16{\pm}0.28$ (kPa), $\mu_2 = 1.97 \pm 0.30$ (kPa)<br>Fibrotic Liver:<br>$\eta = 14.4 \pm 6.6$ (Pa.s), $\mu_1 = 2.91 \pm 0.84$ (kPa), $\mu_2 = 4.83 \pm 1.77$ (kPa)
(Asbach et al., 2010)	Multifrequency MRE with frequencies of 25.0,37.5, 50.0, and 62.5 Hz	Two-parameter spring pot model	Normal liver: (16 volunteers)<br>$\mu = 2.25 \pm 0.43$ (kPa)<br>Stage F1: (20 patients)<br>$\mu = 2.61 \pm 0.43$ (kPa)<br>Stage F2: (17 patients)<br>$\mu = 3.00 \pm 0.63$ (kPa)<br>Stage F3: (16 patients)<br>$\mu = 3.86 \pm 0.61$ (kPa)<br>Stage F4: (19 patients)<br>$\mu = 5.86 \pm 0.1.22$ (kPa)
(Clarke et al., 2011)	MRE at a vibration frequency of 120 Hz under various levels of static compressive pre-strain up to 30%	Exponential model for large strain:<br>$\mathbf{G}^\star = \mathrm{A}\mathrm{e}^{\mathrm{B}\epsilon_\mathrm{G}}$<br>A and B are the model coefficients	$\mathbf{G}' = 1.54\mathrm{e}^{-2.04\epsilon_\mathrm{G}}$ (kPa)<br>$\mathbf{G}'' = 0.62\mathrm{e}^{-2.71\epsilon_\mathrm{G}}$ (kPa)

Table S6: MRE models description

Author	Techniques	Model	Measured parameter
(Chen et al., 2011)	The MR elastography sequence parameters were as follows: phase offsets, four; motion sensitivity, 10.2 mm/radian; axial imaging plane; superior-inferior motion-sensitizing direction; field of view, 34–44 cm; acquisition matrix, 256 3 96; fractional phase field of view, 0.75–1; flip angle, 30°; one signal acquired; bandwidth, 31.25 kHz; echo time msec/repetition time msec, 24.5/50; section thickness, 10 mm; number of sections, two to four; imaging time, two to four breath holds (about 17 seconds each)	elastic	stiffness:<br>patients with simple steatosis: 2.51 (kPa)<br>patients with inflammation but no fibrosis 3.24 (kPa))<br>patients with hepatic fibrosis 4.16 (kPa)
(Dzyubak et al., 2021)	MRE at continuous acoustic pressure waves generated at 60 Hz by an active driver outside the scanner room	NM	NM
(Eaton et al., 2020)	NM	NM	NM

Table S6: MRE models description

Author	Techniques	Model	Measured parameter
(Garteiser et al., 2012)	MRE at 50 Hz using electro-mechanical transducer	NM	Haemangioma: $\mid \mathbf{G}^\star \mid = 2.31 \pm 0.66$ (kPa), $\mathbf{G}' = 2.12 \pm 0.63$ (kPa) $\mathbf{G}'' = 0.88 \pm 0.31$ (kPa) Focal nodular hyperplasia: $\mid \mathbf{G}^\star \mid = 2.51 \pm 1.03$ (kPa), $\mathbf{G}' = 2.13 \pm 0.69$ (kPa) $\mathbf{G}'' = 1.19 \pm 0.95$ (kPa) Adenoma: $\mid \mathbf{G}^\star \mid = 2.13 \pm 0.70$ (kPa), $\mathbf{G}' = 2.01 \pm 0.63$ (kPa) $\mathbf{G}'' = 0.71 \pm 0.33$ (kPa) Metastasis: $\mid \mathbf{G}^\star \mid = 2.99 \pm 0.76$ (kPa), $\mathbf{G}' = 2.36 \pm 0.5$ (kPa) $\mathbf{G}'' = 1.89 \pm 0.70$ (kPa) Hepatocellular carcinoma: $\mid \mathbf{G}^\star \mid = 3.57 \pm 1.71$ (kPa), $\mathbf{G}' = 2.51 \pm 1.01$ (kPa) $\mathbf{G}'' = 2.36 \pm 1.69$ (kPa) Cholangiocarcinoma: $\mid \mathbf{G}^\star \mid = 3.3 \pm 1.77$ (kPa), $\mathbf{G}' = 1.47 \pm 0.23$ (kPa) $\mathbf{G}'' = 2.80 \pm 2.11$ (kPa)
(Gidener et al., 2020)	NM	NM	NM

Table S6: MRE models description

**Author**	**Techniques**	**Model**	**Measured parameter**
(Godfrey et al., 2012)	The passive driver was connected to an active drive unit that produces low frequency longitudinal pressure waves at 60 Hz. Four separate breath-hold acquisitions were acquired using a phasecontrast gradient-echo sequence synchronised to the active driver unit at four different phase steps	elastic	stiffness:<br>disease (from any aetiology) chronic liver 3.45 (kPa)
(Kamphues et al., 2012)	MRE at four sinusoidal transverse waves with frequencies of 25.0, 37.5, 50.0, and 62.5 Hz within one mechanical excitation. The shear modulus μ was derived by assuming a viscosity η of liver tissue of 7.3 $(\mathrm{Pa.s})$	Spring pot model:<br>$\mathbf{G}^{\star} = \kappa(\mathrm{i}\omega)^{\alpha}$, $\kappa = \mu^{(1-\alpha)}\eta^{\alpha}$.<br>$\kappa$ and α are two independent constants. κ is the fractional element and α is the dimensionless powerlaw exponent	Healthy liver:<br>$\mu_{\mathrm{median}} = 1.99\ (\mathrm{kPa}),\ (\ 1.65 - 2.37\ \mathrm{kPa})$<br>Liver with the general prediction of stage of fibrosis:<br>$\mu_{\mathrm{median}} = 3.66\ (\mathrm{kPa}),\ (\ 1.9 - 6.29\ \mathrm{kPa})$<br>$\alpha_{\mathrm{median}} = 0.25\ (-),\ (0.21 - 0.28)$

Table S6: MRE models description

**Author**	**Techniques**	**Model**	**Measured parameter**
(Klatt et al., 2007)	MRE at four driving frequencies between 25 and 62.5 Hz	Voigt:<br>$\mathbf{G}^{\star}(\omega) = \mu + i\omega\eta$<br>Maxwell:<br>$\mathbf{G}^{\star}(\omega) = \dfrac{i\omega\eta\mu}{\mu + i\omega\eta}$<br>Zener:<br>$\mathbf{G}^{\star}(\omega) = \dfrac{\mu_1\mu_2 + i\omega\eta(\mu_1 + \mu_2)}{\mu_2 + i\omega\eta}$<br>Jeffreys:<br>$\mathbf{G}^{\star}(\omega) = -\omega\eta_1 \dfrac{\omega\eta_2 - i\mu}{\mu + i\omega(\eta_1 + \eta_2)}$<br>Fractional Zener:<br>$\mathbf{G}^{\star}(\omega) = \mu_1 + \dfrac{\mu_2(\frac{\mathrm{i}\omega\eta}{\mu_2})^{\alpha}}{1 + (\frac{\mathrm{i}\omega\eta}{\mu_2})^{\alpha}}$	Voigt:<br>$\eta_{\text{mean}} = 2.8\ (\text{Pa s}),\ \mu_{\text{mean}} = 2.09\ (\text{kPa})$<br>$\chi_{\text{mean}} = 0.31\ (\text{kPa})$<br>Maxvell:<br>$\eta_{\text{mean}} = 21.3\ (\text{Pa s}),\ \mu_{\text{mean}} = 2.52\ (\text{kPa})$<br>$\chi_{mean} = 0.28\ (\text{kPa})$<br>Zener:<br>$\eta_{\text{mean}} = 5.5\ (\text{Pa s}),\ \mu_{1\text{mean}} = 1.36\ (\text{kPa})$<br>$\mu_{2\text{mean}} = 1.86\ (\text{kPa})\ \chi_{\text{mean}} = 0.08\ (\text{kPa})$<br>Jeffreys:<br>$\eta_{1\text{mean}} = 41.6\ (\text{Pa s}),\ \eta_{2\text{mean}} = 1.4\ (\text{Pa s})$<br>$\mu_{\text{mean}} = 2.41\ (\text{kPa})\ \chi_{\text{mean}} = 0.25\ (\text{kPa})$<br>Fractional Zener:<br>$\eta_{\text{mean}} = 6.2\ (\text{Pa s}),\ \mu_{1\text{mean}} = 1.2\ (\text{Pa s})$<br>$\mu_{2\text{mean}} = 3.33\ (\text{kPa})\ \ \alpha_{\text{mean}} = 0.91(-)$<br>$\chi_{\text{mean}} = 0.38\ (\text{kPa})$
(Kruse et al., 2000)	MRE with shear wave frequencies of 75, 100, 150, 200, 250 and 300 Hz by transverse motion of a contact plate connected to an electromechanical actuator. Elastographic imaging of a cube of tissue-simulating material (18% bovine gelatin) was also performed at shear wave frequencies ranging from 100 to 500 Hz	Isotropic Hookean:<br>$\mu = v^2\rho$<br>μ : shear stiffness<br>v : shear wave propagation speed<br>ρ : density	$\mu = 2.73\ (\text{kPa})$<br>$\eta = 10.3\ (\text{Pa s})$

Table S6: MRE models description

Author	Techniques	Model	Measured parameter
(Leclerc et al., 2013)	Fibroscan was performed at 50 Hz and multifrequency MRE experiments were performed at 60, 70, and 80 Hz.	Voigt:<br>$\mathbf{G}^\star = \mu + \mathrm{i}\omega\eta$<br>Spring pot:<br>$\mathbf{G}^\star = \mu^{1-\alpha}\eta^{\alpha}(\mathrm{i}\omega)^{\alpha}$	Voigt:<br>$\eta = 0.8 \pm 0.1$ (Pa s)<br>Spring pot:<br>$\eta = 3.9 \pm 0.7$ (Pa s)
(Leclerc et al., 2015)	MRE at 60 Hz and displacement of the cylindrical pneumatic driver membrane	Isotropic homogeneous elastic:<br>$\mu = \rho\lambda^2 f^2$<br>$\rho = 1000\ (\mathrm{kg/m^3})$<br>$\mu$: shear stiffness<br>λ : wavelength<br>f : wave frequency	$\mu = 4.16 \pm 0.14$ (kPa)
(Lee et al., 2014)	The 60 Hz acoustic wave was used as an excitatory stimulus. A 19-cm-diameter and 1.5-cmthick, cylindrical, passive, longitudinal, shear wave driver was placed against the right chest wall over the liver with the center of the driver at the level of the xiphisternum.	elastic	stiffness:<br>normal liver parenchyma 3.45±0.25 (kPa) (1.38–8.48(kPa))<br>chronic liver diseases 4.28±0.33 (kPa) (1.68–8.48(kPa))

Table S6: MRE models description

Author	Techniques	Model	Measured parameter
(Reiter et al., 2014)	MRE was applied in a large dynamic range from 200 to 1200 Hz. For induced wave imaging, a gradient echo sequence enhanced by sinusoidal motion encoding gradients (MEG) was used. The MEG frequency was adapted to the mechanical vibration frequency f from 200 to 1200 Hz in increments of 100 Hz	Spring pot model:<br>E : Static indentation Young's modulus<br>μ: Dynamic shear modulus<br>α: Powerlaw exponent according to the spring pot model	$\mathrm{E_{mean}} = 5.75$ (kPa)<br>$\mu_{mean} = 7.5$ (kPa)<br>$\alpha_{mean} = 0.150$ (rad)<br>$\mathbf{G}' = 8.7$ (kPa)<br>$\mathbf{G}'' = 1.9$ (kPa)
(Riek et al., 2011)	A FLASH sequence was customized for MRE by sinusoidal motion sensitizing gradients (MSG) in the through-plane direction. The MSG strength was 285 mT/m, with frequencies 100–800 Hz matched to the mechanical vibration	Spring pot:<br>$\mathbf{G}^{\star} = \mu^{1-\alpha}\eta^{\alpha}(\mathrm{i}\omega)^{\alpha}$	Fibrotic human liver:<br>$\mu = 57.5$ (kPa), $\alpha = 0.34$<br>Bovine liver:<br>$\mu = 3.7 \pm 0.6$(kPa), $\alpha = 0.28 \pm 0.01$
(Ronot et al., 2014)	The MRE acquisitions were obtained sequentially with three different mechanical excitation frequencies of 500, 600, and 700 Hz	NM	NM

Table S6: MRE models description

Author	Techniques	Model	Measured parameter
(Salameh et al., 2007)	Longitudinal mechanical waves of 200 Hz were transmitted into the liver with a transducer consisting of a coil driven by a programmable pulse generator	Voigt	Control rats:<br>$\mu_{\text{mean}} = 1.76 \pm 0.37$ (kPa)<br>$\eta_{\text{mean}} = 0.51 \pm 0.04$ (kPa)<br>Rats with fibrosis:<br>$\mu_{\text{mean}} = 2.29 \pm 0.32$ (kPa)<br>$\eta_{\text{mean}} = 0.69 \pm 0.12$ (kPa)
(Salameh et al., 2009)	Longitudinal mechanical waves of 300 Hz were transmitted to the liver with a custom-built transducer consisting of two piezoelectric plates driven by a programmable pulse generator	NM	Control rats:<br>$\mu_{\text{mean}} = 1.82 \pm 0.22$ (kPa)<br>$\eta_{\text{mean}} = 0.59 \pm 0.12$ (kPa)<br>Choline-deficient rats:<br>at two weeks<br>$\mu_{\text{mean}} = 2.24 \pm 0.19$ (kPa)<br>$\eta_{\text{mean}} = 0.86 \pm 0.10$ (kPa)<br>at five weeks<br>$\mu_{\text{mean}} = 2.72 \pm 0.45$ (kPa)<br>$\eta_{\text{mean}} = 1.08 \pm 0.20$ (kPa)<br>at eight weeks<br>$\mu_{\text{mean}} = 2.90 \pm 0.49$ (kPa)<br>$\eta_{\text{mean}} = 1.14 \pm 0.19$ (kPa)<br>Orotic acid diet group:<br>$\mu_{\text{mean}} = 2.10 \pm 0.15$ (kPa)<br>$\eta_{\text{mean}} = 0.77 \pm 0.11$ (kPa)<br>Group injected with carbon tetrachloride:<br>$\mu_{\text{mean}} = 2.96 \pm 0.63$ (kPa)<br>$\eta_{\text{mean}} = 0.85 \pm 0.22$ (kPa)

Table S6: MRE models description

Author	Techniques	Model	Measured parameter
(Tang and Wan, 2014)	A simple-to-build data acquisition system for capturing soft-tissue deformations ex vivo, which was used to record the indentation and stretch tests on ex vivo samples	Neo-Hookean: $W = \frac{\mu}{2}(\mathrm{I}_1 - 3) - \mu\log\ \mathrm{J} + \frac{\beta}{2}(\log\ \mathrm{J})^2$ μ and β: Lamé constants $\mu = \frac{\mathrm{E}}{2(1+\nu)},\ \beta = \frac{\mathrm{E}\nu}{(1+\nu)(1-2\nu)}$	$\mathrm{E} = 15\ (\mathrm{kPa})$ $\nu = 0.45\ (-)$
(Tomita et al., 2018)	The MRE acquisitions were obtained with three different frequencies of 62.5, 125, and 250 Hz	Zener: $\mathbf{G}' = \mu_0 + \frac{\mu_1(\omega\eta_1)^2}{\mu_1^2 + (\omega\eta_1)^2}$ $\mathbf{G}'' = \frac{\mu_1(\omega\eta_1)^2}{\mu_1^2 + (\omega\eta_1)^2}$	$\mathbf{G}'$: $62.5\ (\mathrm{Hz}) : 14.5\ (\mathrm{kPa})$ $125\ (\mathrm{Hz}) : 14.9\ (\mathrm{kPa})$ $250\ (\mathrm{Hz}) : 15.0\ (\mathrm{kPa})$
(Tzschätzsch et al., 2014)	Time-harmonic multifrequency MRE	Elastic: $\mathrm{c_M}(\omega_\mathrm{n}) = \sqrt{\frac{\mu}{\rho}}$ Kelvin-Voigt: $\mathrm{c_M}(\omega_\mathrm{n}) = \sqrt{\frac{2[\mu^2 + (\omega\eta)^2]}{\rho[\mu + \sqrt{\mu^2 + (\omega\eta)^2}]}}$	Elastic: THE: $\mu = 1.95\ (\mathrm{kPa})$, MRE: $\mu = 2.23\ (\mathrm{kPa})$ Kelvin-Voigt: THE: $\mu = 1.05\ (\mathrm{kPa})\ \eta = 4.8 (\mathrm{Pa\,s})$ MRE: $\mu = 1.21\ (\mathrm{kPa}),\ \eta = 4.7 (\mathrm{Pa\,s})$
(Wang et al., 2011)	Continuous acoustic vibration at 60 Hz transmitted from an active driver to the passive driver through a flexible vinyl tube was used to produce propagating shear waves in the liver	elastic	stiffness: liver without fibrosis $3.16\ (\mathrm{kPa})\quad (2.62–3.58 (\mathrm{kPa}))$ liver with any degree of fibrosis $6.37\ (\mathrm{kPa})\quad (4.73–8.12 (\mathrm{kPa})$

NM: Not mentioned

Appendix H: Detailed of study population and diseases

Table S7: Detailed of study population and diseases

Author	Study type	Study population characteristic	studied disease
(Amili et al., 2019)	In vitro	Phantom	-
(Asbach et al., 2008)	In vivo	Eight healthy volunteers and eight patients with biopsy-proven liver fibrosis (grade 3–4)	Fibrosis (grade 3–4)
(Asbach et al., 2010)	In vivo	16 healthy volunteers and 72 patients,<br>stage F1: n = 20, stage F2: n = 17, stage F3: n = 16, stage F4: n = 19	Fibrosis (grade 1–4)
(Brock et al., 2005)	In vivo	Five healthy women volunteers, with average age of 33 years (range: 25 to 49)	-
(Chen et al., 2011)	In vivo	A total of 58 subjects (mean (SD) age: 51.5 (25–78 years), BMI: 38.3 (21.2–50.6), (83% female)	Nonalcoholic steatohepatitis in patients with nonalcoholic fatty liver disease
(Clarke et al., 2011)	In vivo	Fresh bovine liver	-
(Courtecuisse et al., 2014)	In vivo	Female pig	-
(Dzyubak et al., 2021)	In vivo	A total of 83 subjects (mean (SD) age: 47 (±11), BMI: 47 (±9), 83% female) from their cohort with successful biopsy and MRE and CSE-MRI	Nonalcoholic steatohepatitis

Table S7: Detailed of study population and diseases

Author	Study type	Study population characteristic	studied disease
(Eaton et al., 2020)	In vivo	a retrospective review of 204 patients with patients who underwent 2 MREs at a single center between January 1, 2007 and December 31, 2018, age: 47 (34-61), BMI: 25.60 (22.90-28.90), 33.44 (69/2014) % female)	Hepatic decompensation in patients with primary sclerosing cholangitis
(Garteiser et al., 2012)	In vivo	72 patients including 27 men with an average age of 59 years (range 20 to 78 years) and 45 women with an average age of 46 years (range 20 to 70 years)	Liver lesions due to tumor
(Gidener et al., 2020)	In vivo	A total of 829 NAFLD subjects (54% women, median age 58 years)	NAFLD
(Godfrey et al., 2012)	In vivo	77 patients (55 male, 22 female) were referred for liver biopsy. The mean age was 49 ± 11.5 years (24–79 years).	Fibrosis
(Hariharan et al., 2007)	In vitro	Excised porcine liver	Tumor ablation
(Hudert et al., 2019)	In vivo	Fifty subjects, F0 (fifteen men, Age: 15.3±1.4 years, BMI: 34.2±5.5 $(\mathrm{kg/m^2})$), F1 (seven men, five women, Age: 14.3±2.3 years, BMI: 36.2±4.6 $(\mathrm{kg/m^2})$), F2 (eight men, one woman, Age: 13.6±2.4 years, BMI: 33.3±5.4 $(\mathrm{kg/m^2})$), F3 (10 men, four women, Age: 13.1±2.0 years, BMI: 31.9±6.1 $(\mathrm{kg/m^2})$)	NAFLD
(Idkaidek and Jasiuk, 2015)	In vitro	Porcine liver	-

Table S7: Detailed of study population and diseases

Author	Study type	Study population characteristic	studied disease
(Kamphues et al., 2012)	In vivo and in vitro	25 patients with liver-transplant	Hepatitis C
(Klatt et al., 2007)	In vivo	Five healthy men volunteers aged 25, 34, 35, 37 and 46 years	-
(Kruse et al., 2000)	In vivo	Juvenile porcine hepatic and renal parenchymal	-
(Lara et al., 2011)	In vitro	Phantom	-
(Leclerc et al., 2013)	In vitro	40 subjects, 10 healthy volunteers (seven men, three women, mean age, 41 years, range 23.8 to 48.4 years) without liver damage, and 30 alcoholic patients (23 men, seven women, mean age, 43 years, range 29.6 to 59.8 years)	Alcoholic liver fibrosis
(Leclerc et al., 2015)	In vitro	A homogeneous phantom composed of 45% softener and 55% liquid plastic	-
(Lee et al., 2010)	In vivo	A normal female subject, four patients (three men, one woman, mean age 66 $\pm$ 8) and a silicone model of the internal organs	-
(Lee et al., 2014)	In vivo	94 consecutive patients (64 males and 30 females; age range, 27–82 years; mean age, 58 years; BMI:, 16.31–31.21 kg/m2	Fibrosis

Table S7: Detailed of study population and diseases

**Author**	**Study type**	**Study population characteristic**	**studied disease**
(Lu and Untaroiu, 2014)	In vivo	15 subjects including six women (height range 1.5 to 1.74, weight range 48 to 91.7, age range 24 to 41) and nine men (height range 1.6 to 1.91, weight range 64 to 102.1, age range 26 to 32)	-
(Ma et al., 2019)	Ex vivo	Living liver donor	-
(Monti et al., 2014)	In vivo	NM	-
(Motosugi et al., 2019)	In vivo	There were 23 participants (mean age, 52.3 years, age range 25 to 75 years), including 14 men (mean age, 51.7 years, age range 25 to 75 years) and nine women (mean age, 53.2 years, age range 31 to 72 years) with no varices (n = 8), low-risk varices (n = 8), and high-risk varices (n = 7) determined at endoscopy	Gastroesophageal varices in patients with liver cirrhosis
(Ning et al., 2018)	In vivo	13 domestic pigs (all female, mean weight 54 kg)	Portal vein embolization
(Reiter et al., 2014)	Ex vivo	17 samples, 16 from human liver tissue and one from fresh bovine liver	Fibrosis
(Reiter et al., 2018)	In vivo	16 healthy volunteers, 15 patients with liver fibrosis in patients with alpha1-antitrypsin deficiency patients (11 homozygous PiZZ, 4 heterozygous PiMZ)	alpha1-antitrypsin deficiency

Table S7: Detailed of study population and diseases

Author	Study type	Study population characteristic	studied disease
(Reiter et al., 2020)	In vivo	16 healthy volunteers (eight men and eight women) and 45 patients (27 men and 18 women), Patients and healthy volunteers had a mean age of 49 years (range 16 to 75 years) and 52 years (range 31 to 75 years)	Fibrosis
(Riek et al., 2011)	Ex vivo	Fresh bovine liver, bovine muscle, and calf brain	-
(Roldán-Alzate et al., 2013)	In vivo	17 patients (58.6 $\pm$ 6.73 years, 88.4 $\pm$ 6.7 kg, 13 men, four women) with portal hypertension and seven (32.2 $\pm$ 10.1 years, 85.7 $\pm$ 8.7 kg, four men, three women) subjects with no liver disease	Cirrhosis and portal hypertension
(Ronot et al., 2014)	Ex vivo	50 male Wistar rats aged eight weeks and weighting 252$\pm$ 28 g. Eight rats were used as controls, and liver fibrosis was induced in the 42 other rats	Fibrosis
(Rutkowski et al., 2018)	In vivo and in vitro	Three healthy subjects with no known liver disease	Liver transplant
(Rutkowski et al., 2019)	In vivo	12 subjects, six with cirrhosis and six with no known liver disease	Cirrhosis
(Salameh et al., 2007)	In vivo	15 adult male Wistar rats weighing 386 $\pm$ 9 g, five controls and 10 rats with liver fibrosis induced by intraperitoneal injections of carbon tetrachloride	Fibrosis

Table S7: Detailed of study population and diseases

Author	Study type	Study population characteristic	studied disease
(Salameh et al., 2009)	In vivo	55 male Sprague-Dawley rats (mean weight: 268 g $\pm$ 53, mean age: nine weeks$\pm$2), 12 control and 24 rats with fatty liver	Steatohepatitis in fatty liver
(Shahryari et al., 2019)	In vivo	Seven healthy volunteers and 70 patients with a total of 105 malignant and 36 benign lesions	Liver lesions of different etiologies
(Stoter et al., 2017)	In vivo	NM	-
(Tang and Wan, 2014)	In vitro	Porcine liver	-
(Tomita et al., 2018)	In vitro	Agarose gel phantom, a healthy volunteer (man, age 22 years)	-
(Tzschätzsch et al., 2014)	In vivo	Eight healthy volunteers (mean age, 35 years, range, 27 to 52 years) and in a patient with biopsy-proven cirrhosis	Liver fibrosis

Table S7: Detailed of study population and diseases

Author	Study type	Study population characteristic	studied disease
(Wang et al., 2011)	In vivo	76 patients (50 men and 26 women; median age, 55 years; (20–74 years)	viral hepatitis in 47 patients (chronic hepatitis C in 44 patients, chronic hepatitis B in two patients, and chronic hepatitis C combined with alcohol abuse in one patient). Nonalcoholic steatohepatitis in five patients, nonalcoholic steatosis in three patients, autoimmune diseases in nine (autoimmune hepatitis in four patients and primary sclerosing cholangitis in five), Wilson disease in one, cystic fibrosis in one, heavy alcohol abuse in one, and nonspecific chronic liver disease in nine patients.
(Zhang et al., 2013)	In vivo	11 healthy volunteers	-
(Zhang et al., 2014)	In vivo	A patient with liver tumor	Liver tumor

-: The paper studied healthy liver, experimental model or phantom, NM: Not mentioned

Appendix I: Quality Assessment

Table S8: Quality Assessment

Author	1	2	3	4	5	6	7	8	9	10	11	12	Quality
(Amili et al., 2019)	1	1	1	NA	0	NA	1	0	1	1	1	1	High
(Asbach et al., 2008)	1	1	1	2	2	1	1	1	1	1	0	0	High
(Asbach et al., 2010)	1	0	1	2	2	1	1	1	1	1	1	0	High
(Brock et al., 2005)	1	1	1	2	2	1	1	1	1	0	1	0	High
(Clarke et al., 2011)	1	1	1	NA	NA	1	1	0	1	0	1	1	High
(Chen et al., 2011)	1	1	1	2	2	1	1	1	1	1	1	1	High
(Courtecuisse et al., 2014)	1	0	0	NA	0	0	1	0	1	0	0	0	Low
(Dzyubak et al., 2021)	1	1	1	1	1	1	1	1	1	1	0	1	High
(Eaton et al., 2020)	1	1	1	1	1	1	1	1	1	1	0	1	High
(Garteiser et al., 2012)	1	0	1	2	2	1	1	1	1	1	0	0	High
(Gidener et al., 2020)	1	1	1	1	1	1	1	1	1	1	0	1	High
(Godfrey et al., 2012)	1	0	1	2	2	1	1	1	1	1	1	0	High
(Hariharan et al., 2007)	1	0	0	0	0	0	1	0	1	1	1	0	Low
(Hudert et al., 2019)	1	0	1	2	2	1	1	1	NA	1	1	1	High
(Idkaidek and Jasiuk, 2015)	1	0	1	NA	0	1	1	0	1	0	0	1	Low
(Kamphues et al., 2012)	1	1	1	2	2	1	1	1	1	1	0	0	High
(Klatt et al., 2007)	1	0	1	2	2	1	1	1	1	1	0	1	High
(Kruse et al., 2000)	1	1	1	NA	0	0	1	0	1	1	1	0	High
(Lara et al., 2011)	1	0	1	0	0	0	1	1	1	1	1	0	Low
(Leclerc et al., 2013)	1	0	1	2	2	1	1	1	1	0	0	0	High
(Leclerc et al., 2015)	1	0	1	2	2	1	1	1	1	0	0	0	High
(Lee et al., 2010)	1	0	1	2	1	0	1	1	1	0	0	0	High
(Lee et al., 2014)	1	0	1	2	2	1	1	1	1	0	1	0	High
(Lu and Untaroiu, 2014)	1	0	1	2	2	1	1	0	NA	1	0	1	High
(Ma et al., 2019)	1	0	1	0	0	0	1	1	1	1	1	0	Low
(Monti et al., 2014)	1	0	0	0	0	0	1	0	1	1	1	1	Low
(Motosugi et al., 2019)	1	1	1	2	2	1	1	1	NA	1	1	1	High
(Ning et al., 2018)	1	0	1	NA	2	1	1	0	NA	1	1	0	High
(Reiter et al., 2014)	1	1	1	0	2	1	1	0	1	0	1	1	High
(Reiter et al., 2018)	1	0	1	2	2	1	1	1	0	1	1	1	Medium
(Reiter et al., 2020)	1	0	1	2	2	1	1	1	0	1	1	1	Medium
(Riek et al., 2011)	1	0	1	NA	0	1	1	0	1	0	1	1	Low
(Roldán-Alzate et al., 2013)	1	0	1	2	2	1	1	1	NA	1	1	0	High
(Ronot et al., 2014)	1	0	1	NA	2	1	1	0	1	1	1	1	High
(Rutkowski et al., 2018)	1	0	1	0	1	1	1	1	NA	0	1	1	Medium
(Rutkowski et al., 2019)	1	1	1	2	2	1	1	1	1	1	1	1	High
(Salameh et al., 2007)	1	0	1	NA	2	1	1	1	1	1	1	1	High
(Salameh et al., 2009)	1	0	1	NA	2	1	1	1	1	1	1	0	High
(Shahryari et al., 2019)	1	1	1	2	2	1	1	1	NA	1	1	1	High
(Stoter et al., 2017)	1	0	1	0	0	0	1	1	1	1	1	0	Low

Table S8: Quality Assessment

Author	1	2	3	4	5	6	7	8	9	10	11	12	Quality
(Tang and Wan, 2014)	1	0	1	0	0	1	1	0	1	0	0	0	Low
(Tomita et al., 2018)	1	0	1	2	0	1	1	1	1	0	1	0	Low
(Tzschätzsch et al., 2014)	1	0	1	1	2	0	1	1	1	1	0	0	High
(Wang et al., 2011)	1	0	1	1	2	2	1	1	1	1	0	0	High
(Zhang et al., 2013)	1	1	1	0	2	1	1	1	1	1	0	0	High
(Zhang et al., 2014)	1	0	1	0	0	0	1	0	1	1	0	0	Low

NA: Not applicable or Not mentioned
